# Dexmedetomidine and argon in combination against ferroptosis through tackling TXNIP-mediated oxidative stress in DCD porcine livers

**DOI:** 10.1038/s41420-024-02071-7

**Published:** 2024-07-11

**Authors:** Qian Chen, Jiashi Sun, Xiangfeng Liu, Zhigang Qin, Jieyu Li, Jianbo Ma, Zhengwei Xue, Yirong Li, Ziheng Yang, Qizhe Sun, Lingzhi Wu, Enqiang Chang, Hailin Zhao, Yiwen Zhang, Jianteng Gu, Daqing Ma

**Affiliations:** 1 Department of Anesthesiology, Perioperative and Systems Medicine, Children’s Hospital, Zhejiang University School of Medicine, National Clinical Research Centre for Child Health, Hangzhou, Zhejiang China; 2 Department of Anesthesiology, Southwest Hospital, Army Medical University, Chongqing, China; 3 Division of Anaesthetics, Pain Medicine and Intensive Care, Department of Surgery and Cancer, Faculty of Medicine, Imperial College London, Chelsea & Westminster Hospital, London, UK; 4 Department of Anesthesiology, Shunde Hospital, Southern Medical University, Shunde, Guangdong China

**Keywords:** Translational research, Preclinical research

## Abstract

Graft availability from donation after circulatory death (DCD) is significantly limited by ischaemia reperfusion (IR) injury. Effective strategies to mitigate IR injury in DCD grafts are essential to improve graft quality and expand the donor pool. In this study, liver grafts from DCD pigs were preserved in the University of Wisconsin (UW) solution saturated with 0.1 nM dexmedetomidine (Dex) and various concentrations of noble gases Argon (Ar) and/or Xenon (Xe) at 4 °C for 24 or 72 h. The combined 50% Ar and Dex provided maximum protection to liver grafts by reducing morphological damage, apoptosis, necroptosis, ferroptosis, hepatocyte glycogen depletion, reticulin framework collapse, iron deposition, and oxidative stress. In vitro, human liver Hep G2 cells were preserved in the UW solution saturated with 0.1 nM Dex and 50% Ar in combination at 4 °C for 24 h, followed by recovery in medium at 37 °C for up to 48 h to mimic clinical IR injury. This treatment significantly increased the expression of anti-oxidative stress proteins by promoting the translocation of thioredoxin-interacting protein (TXNIP) to mitochondria, thereby inhibiting ferroptosis, increasing plasma membrane integrity, and maintaining cell viability.In summary, The combination of 0.1 nM Dex and 50% Ar may be a promising strategy to reduce ferroptosis and other form cell death, and preserve liver grafts.

## Introduction

Due to advancements in surgical techniques, immunosuppressant developments, and post-operative care, the success rate of liver transplantation has significantly increased [1]. However, the demand for liver grafts greatly exceeds the available supply, resulting in lengthy waiting lists for patients requiring a liver transplant. In 2019, approximately 118,000 patients were waiting for an organ transplant, with the majority of them awaiting a kidney or liver [2]. Efforts are underway to expand the availability of liver donor organs, with a strategy being the increase in donation after circulatory death (DCD) donors [3].

There is a growing understanding of how mitochondrial metabolism contributes to ischaemia reperfusion (IR) injury and poor outcomes in liver transplantation. Due to the imbalance between the production of reactive oxygen species (ROS) and antioxidant defence mechanisms, mitochondrial oxidative stress poses a significant challenge in liver transplantation [4]. Oxidative stress negatively affects various cellular components, including membranes, lipids, lipoproteins, and deoxyribonucleic acid (DNA) [5, 6], initiating signalling cascades that activate regulated cell death pathways, ultimately damaging liver grafts [7–9].

The supplementation of noble gases such as xenon (Xe) or argon (Ar) during cold preservation has been proposed as a strategy to reduce IR injury [10, 11]. Dexmedetomidine (Dex), a selective α_2_-adrenergic receptor agonist with a sedative effect, has been reported to mitigate IR injury by reducing oxidative stress and inflammatory responses [12, 13]. Accordingly, in this study, we investigated whether Ar, Xe, or Dex, alone or in combination, provided increased protection against IR injury, thereby enhancing liver graft quality. The underlying molecular mechanisms were also explored.

## Results

### Dex and noble gases improved the morphology of cold-preserved DCD livers

**Fig. 1:**
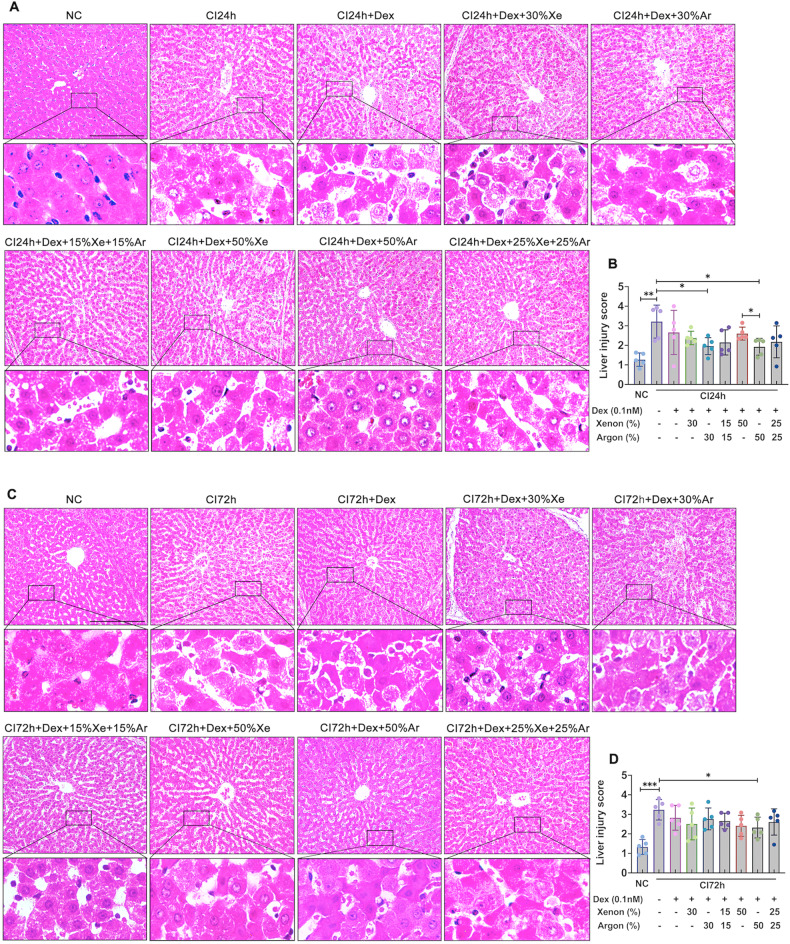
Dex and noble gases protected DCD liver morphology after cold preservation. Livers from experimental mini-pig were harvested 30 min after circulatory death and then stored at 0–4 °C in the UW solution with 0.1 nM Dex plus varying concentrations of Xe and Ar for 24 (CI24h) or 72 h (CI72h). Control tissues (NC) were fixed upon procurement without cold preservation. **A** H&E staining of the liver cold stored for 24 h with Dex or noble gases Xe or/and Ar. **B** 24 h of liver injury score. **C** H&E staining of the liver cold stored for 72 h with Dex or noble gases Xe or/and Ar. **D** 72 h of liver injury score. The liver injury criteria were evaluated with cell disruption, nuclear loss, and tissue structure damage. Scores 0: normal, 1: <25%, 2: 25–50%, 3: 50–75%, 4: > 75% percentage area. Data were analysed with unparied t-test and are presented as scatter plots with bar, mean ± SD. *n* = 5. Scale bar = 200 µm. *p < 0.05, **p < 0.01, ***p < 0.001. DCD Donation after circulatory Death, Dex Dexmedetomidine, Xe Xenon, Ar Argon, CI cold ischaemia, NC negative control.

When compared to non-cold preserved negative controls (NC), 24- or 72-h cold-preserved livers (CI24h or CI72h) exhibited significant morphological changes, including cellular swelling, plasma membrane rupture, nuclei breakdown and loss of staining (Fig. 1A, C). After 24 h cold preservation, the liver injury score increased to 3.21 ± 0.84 (*p* < 0.01). However, Dex combined with 30% or 50% Ar significantly reduced hepatocyte necrosis and decreased liver injury score to 1.96 ± 0.43 (*p* < 0.05) or 1.93 ± 0.43 (*p* < 0.05), respectively (Fig. 1B). After 72 h of cold preservation, the liver injury score rose to 3.23 ± 0.52 (*p* < 0.001); Dex combined with 50% Ar significantly preserved hepatocytes and reduced the liver injury score to 2.32 ± 0.53 (*p* < 0.05, Fig. 1D).

### Dex and noble gases reduced apoptosis in cold-preserved DCD livers

**Fig. 2:**
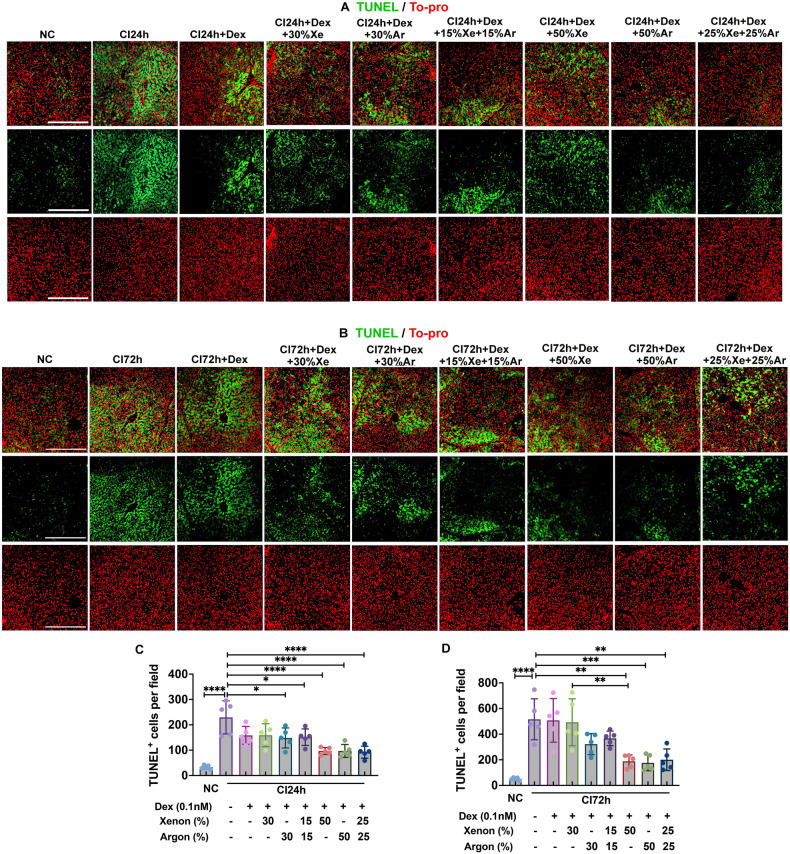
Dex and noble gases reduced apoptosis in DCD livers. Livers from experimental mini-pig were harvested 30 min after circulatory death and then stored at 0–4 °C in the UW solution with 0.1 nM Dex plus varying concentrations of Xe and Ar for 24 (CI24h) or 72 h (CI72h). Control tissues (NC) were fixed upon procurement without cold preservation. **A** TUNEL (green) and To-Pro (red) staining in the liver upon 24 h of cold preservation. **B** Average number of TUNEL^+^ apoptotic cells per field of view upon 24 h of cold preservation. **C** TUNEL (green) and To-Pro (red) staining in the liver upon 72 h of cold preservation. **D** Average number of TUNEL^+^ apoptotic cells per field of view upon 72 h of cold preservation. Data were analysed by one-way ANOVA following Tukey multiple comparisons and are presented as scatter plot with bar, mean ± SD. *n* = 5. Scale bar = 500 µm. *p < 0.05, **p < 0.01, ***p < 0.001, ****p < 0.0001. DCD Donation after circulatory Death, Dex Dexmedetomidine, Xe Xenon, Ar Argon, CI cold ischaemia, NC negative control, TUNEL Terminal deoxynucleotidyl transferase dUTP nick end labelling.

The average number of TUNEL^+^ apoptotic cells significantly increased from 33.04 ± 7.43 (NC) to 229.41 ± 65.28 cells per field after 24 h of cold preservation (*p* < 0.0001, Fig. 2A, C), and from 51.22 ± 10.58 (NC) to 515.84 ± 159.71 cells per field after 72 h of cold preservation (*p* < 0.0001, Fig. 2B, D). Significant decreases in TUNEL^+^ cells at 24 h were observed when cold preservation solutions were supplemented with Dex combined with 30% Ar (*p* < 0.05), 15% Xe and 15% Ar, (*p* < 0.05), 50% Xe (*p* < 0.0001), 50% Ar (*p* < 0.0001), or 25% Xe and 25% Ar (*p* < 0.0001) (Fig. 2C). After 72 h, significant reductions in TUNEL^+^ cells were noted with Dex combined with 50% Xe (*p* < 0.01), 50% Ar (*p* < 0.001), or 25% Xe and 25% Ar (*p* < 0.01) (Fig. 2D).

### Dex and noble gases reduced necroptosis in cold-preserved DCD livers

**Fig. 3:**
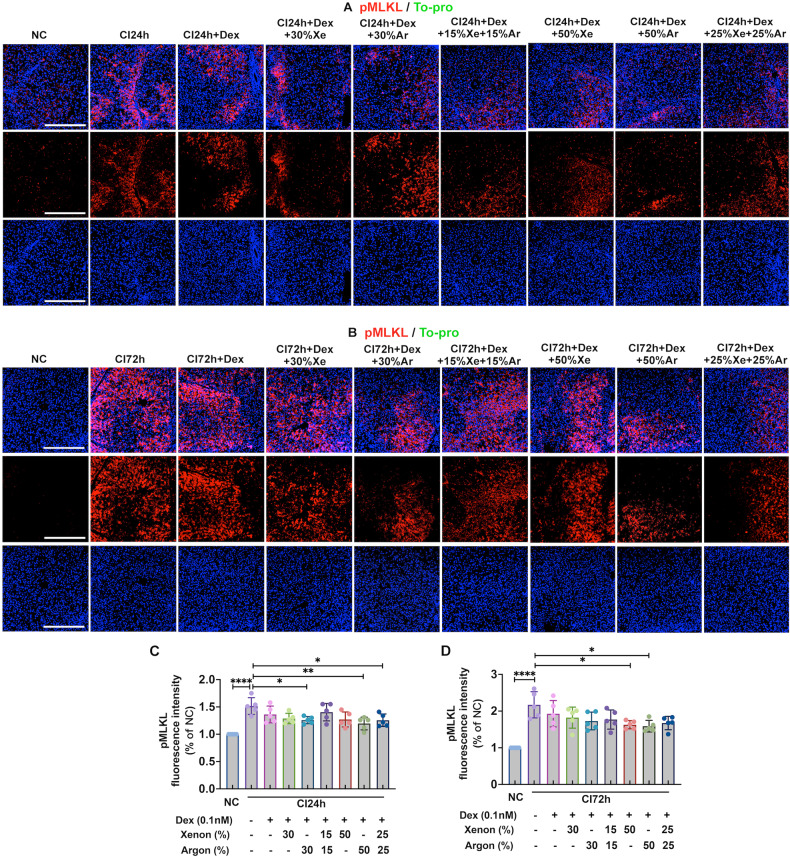
Dex and noble gases reduced necroptosis in DCD livers. Livers from experimental mini-pig were harvested 30 min after circulatory death and then stored at 0–4 °C in the UW solution with 0.1 nM Dex plus varying concentrations of Xe and Ar for 24 (CI24h) or 72 h (CI72h). Control tissues (NC) were fixed upon procurement without cold preservation. **A** pMLKL (red) and DAPI (blue) staining in the liver upon 24 h of cold preservation. **B** Average pMLKL fluorescence intensity per field of view upon 24 h of cold preservation. **C** pMLKL (red) and DAPI (blue) staining in the liver upon 72 h of cold preservation. **D** Average pMLKL fluorescence intensity per field of view upon 72 h of cold preservation. Data were analysed by unpaired t-test and are presented as scatter plots with bar, mean ± SD. *n* = 5. Scale bar = 500 µm. *p < 0.05, **p < 0.01, ****p < 0.0001. DCD Donation after circulatory Death, Dex Dexmedetomidine, Xe Xenon, Ar Argon, CI cold ischaemia, NC negative control, pMLKL phosphorylated Mixed Lineage Kinase domain-like protein, DAPI 4′,6-diamidino-2-phenylindole.

After 24 h of cold preservation, the expression of necroptosis biomarker, pMLKL, significantly increased by 51.6% (*p* < 0.0001), while the solutions supplemented with Dex combined with 30% Ar (*p* < 0.05), 50% Ar (*p* < 0.01), or 25% Xe and 25% Ar (*p* < 0.05) decreased pMLKL expression (Fig. 3A, C). After 72 h of cold preservation, pMLKL expression increased 2.1-fold (*p* < 0.0001), while supplementation with 50% Xe or 50% Ar significantly reduced pMLKL expression (*p* < 0.05, separately; Fig. 3B, D).

### Dex and noble gases reduced ferroptosis in cold-preserved DCD livers

**Fig. 4:**
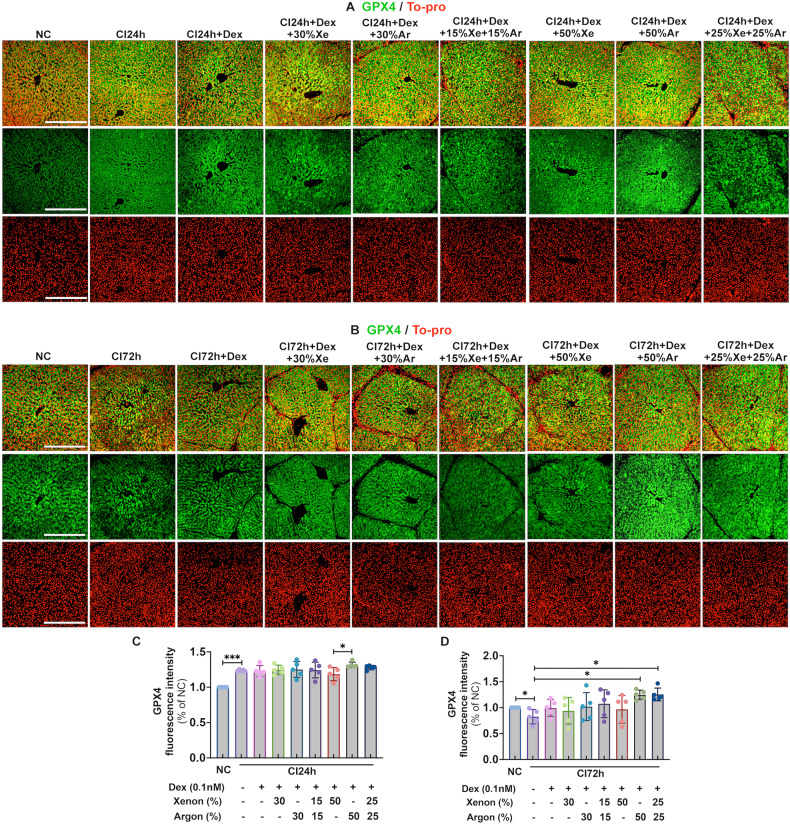
Dex and noble gases reduced ferroptosis in DCD livers. Livers from experimental mini-pig were harvested 30 min after circulatory death and then stored at 0–4 °C in the UW solution with 0.1 nM Dex plus varying concentrations of Xe and Ar for 24 (CI24 h) or 72 h (CI72h). Control tissues (NC) were fixed upon procurement without cold preservation. **A** GPX4 (green) and To-pro (red) staining in the liver upon 24 h of cold preservation. **B** Average GPX4 fluorescence intensity per field of view upon 24 h of cold preservation. **C** GPX4 (green) and To-pro (red) staining in the liver upon 72 h of cold preservation. **D** Average GPX4 fluorescence intensity per field of view upon 72 h of cold preservation. Data were analysed by unpaired t-test and are presented as scatter plots with bar, mean ± SD. *n* = 5. Scale bar = 500 µm. *p < 0.05, ***p < 0.001. DCD Donation after circulatory Death, Dex Dexmedetomidine, Xe Xenon, Ar Argon, CI cold ischaemia, NC negative control, GPX4 Glutathione peroxidase 4.

Cold preservation significantly increased GPX4 expression at 24 h (*p* < 0.001, Fig. 4A, C). While 72 h of cold preservation significantly decreased GPX4 expression (*p* < 0.05, Fig. 4B, D), demonstrating the occurrence of ferroptosis. In addition, Dex combined with 50% Ar (*p* < 0.05) or 25% Xe and 25% Ar (*p* < 0.05) significantly reduced ferroptosis by increasing GPX4 expression (Fig. 4D).

### Dex and noble gases reduced glycogen depletion, reticulin framework collapses, and iron deposition in cold-preserved DCD livers

**Fig. 5:**
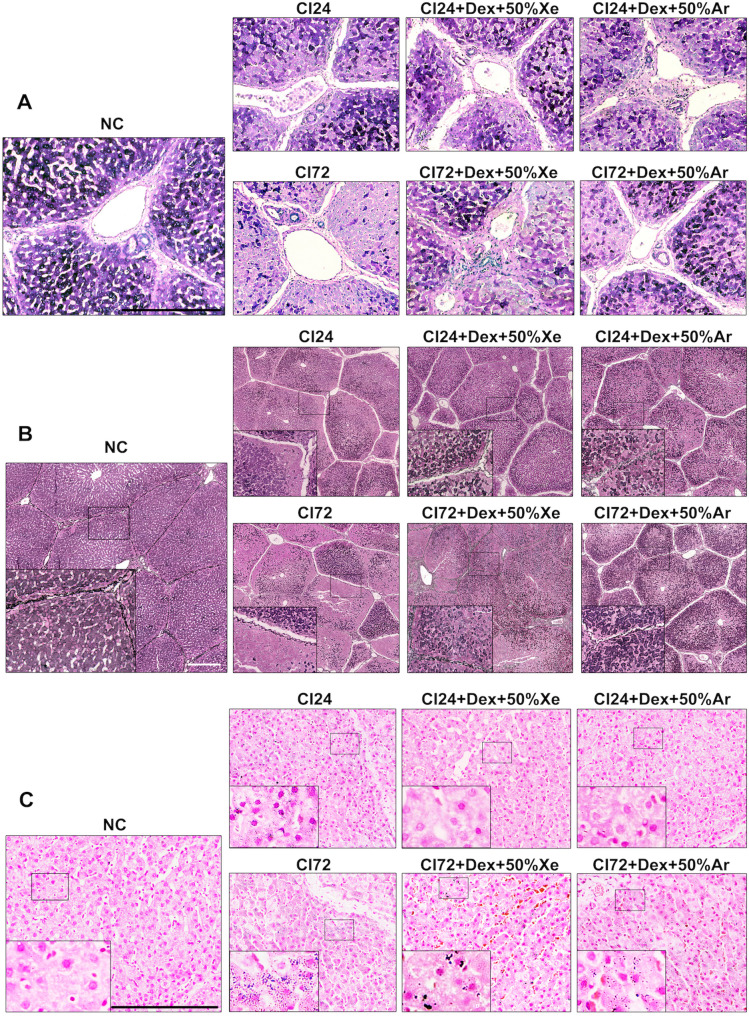
Dex and noble gases reduced glycogen depletion, reticulin framework collapses, and iron deposition in cold-preserved DCD livers. Livers from experimental mini-pig were harvested 30 min after circulatory death and then stored at 0–4 °C in the UW solution with 0.1 nM Dex plus 50% of Xe or Ar for 24 (CI24h) or 72 h (CI72h). Control tissues (NC) were fixed upon procurement without cold preservation. **A** PAS staining; **B** reticulum staining; **C** iron staining was conducted in the liver upon 24 or 72 h of cold preservation. Scale bar = 500 µm. DCD Donation after circulatory Death, Dex Dexmedetomidine, Xe Xenon, Ar Argon, CI cold ischaemia, NC negative control, PAS periodic acid-schiff.

PAS staining (Fig. 5A) revealed moderate glycogen depletion after 24 h of cold preservation, indicated by hepatocytes with lighter purple cytoplasm. The glycogen content improved when solutions were supplemented with Dex combined with 50% Xe or 50% Ar. After 72 h, severe glycogen depletion was observed, while Dex combined with 50% Xe or 50% Ar attenuated this depletion. Reticulum staining (Fig. 5B) showed reticular fibre collapse and massive hepatocyte necrosis after 24 and 72 h of cold preservation, which was alleviated by solutions supplemented with Dex combined with 50% Xe or 50% Ar. Iron staining (Fig. 5C) indicated extensive iron deposition in hepatocytes after 24 or 72 h of cold preservation, which was reduced by solutions supplemented with Dex combined with 50% Xe or 50% Ar.

### Dex and noble gases reduced oxidative stress and TXNIP expression in cold-preserved DCD livers

**Fig. 6:**
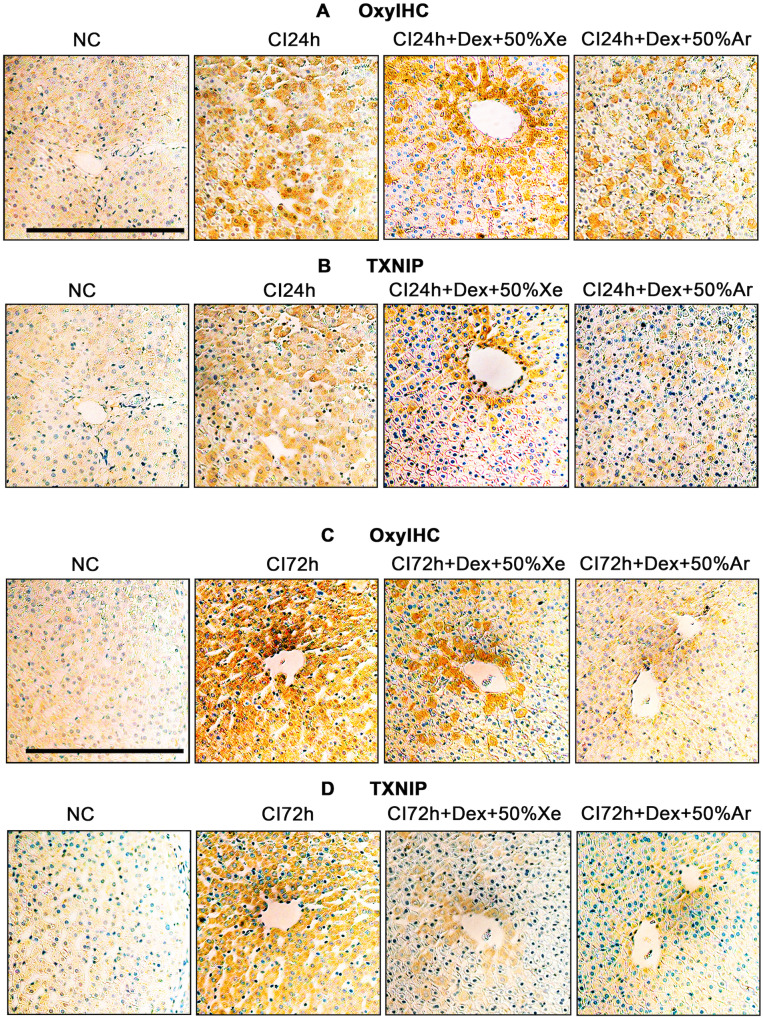
Dex and noble gases reduced oxidative stress and TXNIP expression in DCD livers. Experimental mini-pig livers were harvested 30 min after circulatory death and then stored at 0–4 °C in the UW solution with 0.1 nM Dex plus 50% of Xe and Ar for 24 (CI24h) or 72 h (CI72h). Control tissues (NC) were fixed upon procurement without cold preservation. **A** Oxidative damage in DCD livers cold stored for 24 h was evaluated by OxyIHC oxidative stress detecting assay. **B** TXNIP expression in DCD livers cold stored for 24 h was evaluated by immunochemistry. **C** Oxidative damage in DCD livers cold stored for 72 h was evaluated by OxyIHC oxidative stress detecting assay. **D** TXNIP expression in DCD livers cold stored for 72 h was evaluated by immunochemistry. *n* = 5. Scale bar = 500 µm. DCD Donation after circulatory Death, Dex Dexmedetomidine, Xe Xenon, Ar Argon, CI cold ischaemia, NC negative control, TXNIP thioredoxin-interacting protein.

Both oxidative stress and TXNIP expression increased after 24 and 72 h of cold preservation and were ameliorated by treatment with Dex combined with 50% Xe or 50% Ar (Fig. 6A–D). Notably, the distribution of TXNIP expression overlapped significantly with the location of oxidative stress, indicating a strong relationship between oxidative stress and TXNIP expression.

### Dex and Ar inhibited ferroptosis through anti-oxidative stress in human HepG2 cells

**Fig. 7:**
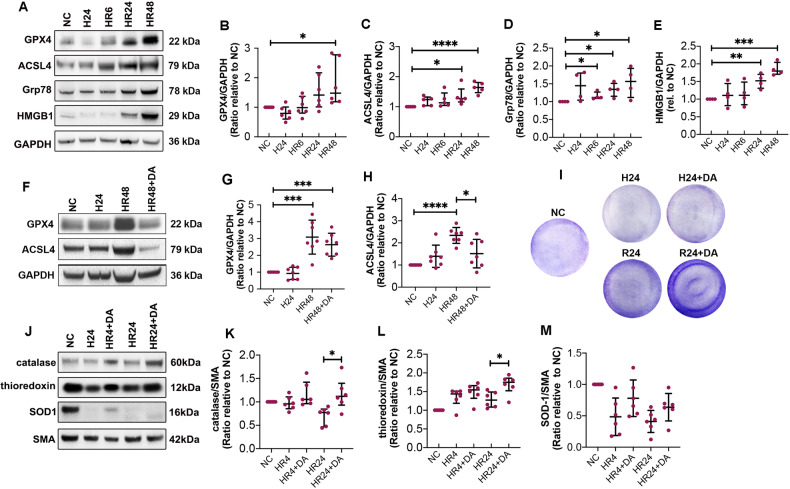
Dex and Ar inhibited ferroptosis through anti-oxidative stress in human HepG2 cells. Human liver HepG2 cells were preserved in 0–4 °C UW solution for 24 h (H24h) and then re-cultured in 37 °C fresh DMEM medium for 6 (HR6), 24 (HR24), and 48 h (HR48). **A** Ferroptosis was evaluated by detecting GPX4 and ACSL4 expression, endothelial reticulum stress was evaluated by detecting Grp78 expression, and Intracellular HMGB1 was evaluated using Western blot. Analysis shows the expression of **B** GPX4, **C** ACSL4, **D** Grp78, and **E** intracellular HMGB1. The cells were re-cultured in 37 °C fresh DMEM medium or in DMEM medium saturated with 0.1 nM Dex combined with 50% Ar for 48 h (HR48 and HR48 + DA) followed by hypothermic hypoxia. **F** Ferroptosis was evaluated by detecting GPX4 and ACSL4 expression. Analysis shows the expression of **G** GPX4, **H** ACSL4, **I** Cell cultures viability was evaluated by crystal violet staining. The cells were re-cultured in 37 °C fresh DMEM medium or DMEM medium saturated with 0.1 nM Dex combined with 50% Ar for 4 (HR4 and HR4 + DA) or 24 h (HR24 and HR24 + DA). **J** Antioxidant proteins catalase, thioredoxin, and SOD1 were detected by Western blot. Analysis shows the expression of **K** catalase, **L** thioredoxin, **M** SOD-1. GAPDH or SMA as a loading control. Data were analysed by unpaired t-test and are presented as scatter plots, median with interquartile range. *n* = 5–7. *p < 0.05, **p < 0.01, ***p < 0.001, ****p < 0.0001. H24 hypothermic hypoxia for 24 h, HR hypothermic hypoxia reoxygenation, DA Dex + Ar, NC negative control, GPX4 Glutathione peroxidase 4, ACSL4 Acyl-CoA Synthetase Long Chain Family Member 4, SOD-1 superoxide dismutase 1, Grp78 Glucose-Regulated Protein 78, HMGB1 High mobility group box 1, GAPDH Glyceraldehyde 3-phosphate dehydrogenase, SMA Smooth muscle actin.

Hypothermic hypoxia reoxygenation (HR) significantly increased GPX4 expression in a time-dependent manner up to 48 h of reoxygenation (*p* < 0.05, Fig. 7A, B). ACSL4 expression also increased at 24 (*p* < 0.05) and 48 h (*p* < 0.0001) (Fig. 7A, C) after reoxygenation, indicating ferroptosis. Grp78 expression, indicating endoplasmic reticulum (ER) stress, increased time-dependently after reoxygenation for 6, 24, and 48 h (*p* < 0.05, respectively; Fig. 7A, D). Intracellular HMGB1 (Fig. 7A, D) also increased after reoxygenation at 24 (*p* < 0.01) and 48 h (*p* < 0.001) (Fig. 7A, E). After 48 h of reoxygenation, the expression of GPX4 (*P* < 0.001) and ACSL4 (*p* < 0.0001) was increased significantly, while Dex and 50% Ar in combination decreased ACSL4 expression (*p* < 0.05, Fig. 7F–H). In addition, crystal violet staining (Fig. 7I) demonstrated that Dex combined with 50% Ar increased cell number and viability after 24 h of hypothermic hypoxia and 24 h of reoxygenation. Antioxidant proteins, catalase, thioredoxin, and SOD-1 were detected to assess the effects of Dex and 50% Ar on early-oxidative stress at 4 h and late-oxidative stress at 24 h of reoxygenation (Fig. 7J). At the late-oxidative stress stage, Dex combined with 50% Ar significantly upregulated the expression of catalase and thioredoxin (*p* < 0.05, respectively; Fig. 7K, L) rather than SOD-1 (Fig. 7M).

### Dex and Ar modulated TXNIP translocation and maintained plasma membrane integrity in HepG2 cells

**Fig. 8:**
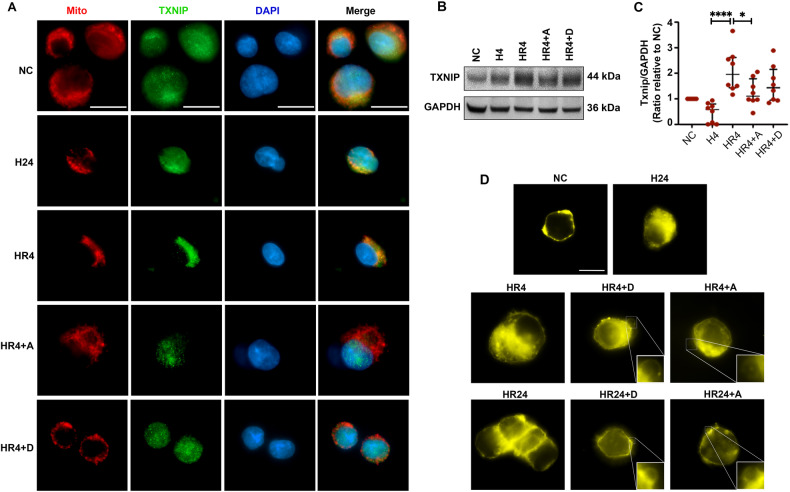
Dex and Ar modulated TXNIP translocation and maintained plasma membrane integrity in HepG2 cells. Human liver HepG2 cells were preserved in 0–4 °C UW solution or UW solution for 24 h (H24h) and then re-cultured in 37 °C fresh DMEM medium or DMEM medium saturated with 0.1 nM Dex (HR + D) or 50% Ar (HR + A) for 4 or 24 h. **A** Mitochondrial (Red) and Txnip (Green) were observed by immunofluorescence. Nuclei were counterstained with 4’,6-diamidino-2-phenylindole (DAPI) (blue). Scale bar: 20 μm. **B** The expression of Txnip was detected by Western blot. Analysis shows the expression of **C** Txnip. GAPDH as a loading control. **D** Plasma membrane was stained with DilC, and the membrane vesicles were captured under an immunofluorescence microscope. Membrane vesicles were framed and enlarged in the lower right corner of the image. Scale bar: 20 μm. Data were analysed by unpaired t-test and are presented as scatter plots, median with interquartile range. *n* = 5–8. *p < 0.05, ****p < 0.0001. H24 hypothermia for 24 h, HR hypothermia reoxygenation, D Dex, A Ar, NC negative control, TXNIP thioredoxin-interacting protein, GAPDH Glyceraldehyde 3-phosphate dehydrogenase.

In HepG2 cells, TXNIP was primarily located in the nuclei and cytoplasm under normal conditions (NC) and after 24 h of hypothermic hypoxia (H24). After 4 h of reoxygenation (HR4), TXNIP translocated to the mitochondria. In contrast, when the UW solution was saturated with 0.1 nM Dex (HR4 + D) or 50% Ar (HR4 + A), TXNIP remained primarily in the nucleus (Fig. 8A). In addition, the expression of TXNIP significantly increased after 4 h of reoxygenation (*p* < 0.0001), but 50% Ar reduced this increase significantly (*p* < 0.05, Fig. 8B, C). Membrane staining (Fig. 8D) showed that, in normal HepG2 cells, the cell membrane was stained bright yellow. Hypothermic hypoxia and reoxygenation increased the permeability of the cell membrane, allowing more DiLC dye to enter the cells and stain the intracellular organelle membranes bright yellow. Membrane vesicles labelled with DilC were found when the UW solution was saturated with Dex or 50% Ar.

## Discussion

The present study demonstrated that DCD porcine livers suffered severe injury after prolonged cold preservation. Supplementation of Dex and noble gases (Xe or Ar) in the UW solution significantly mitigated the activation of regulated cell death signalling pathways, attenuated glycogen depletion, reticulin framework collapse, iron deposition, and reduced oxidative stress. Meanwhile, the UW solution containing 0.1 nM Dex combined with 50% Ar provided a significant protection to maintain liver graft quality during cold preservation. In vitro, HepG2 cells subjected to “hypothermic hypoxia-reoxygenation” experienced ferroptosis; however, treatment with 0.1 nM Dex and 50% Ar significantly increased the expression of antioxidative stress proteins, catalase and thioredoxin, and inhibited TXNIP translocation, thereby preventing ferroptosis and maintaining cell viability. Moreover, Dex and Ar, in combination, repaired the damaged cell membrane by forming membrane vesicles. Collectively, our data suggested that the combination of 0.1 nM Dex and 50% Ar against ferroptosis may present a potential therapeutic strategy to improve the quality of DCD livers for clinical use, although further studies are warranted.

Iron is essential for liver function, contributing to various physiological processes, such as iron storage, heme synthesis, and detoxification [14]. The liver stores about 10% of the body’s iron, while excess free iron catalyses the formation of free radicals *via* the Fenton reaction, leading to oxidative stress and cellular damage [15]. The liver is particularly susceptible to oxidative stress due to its role in metabolism and detoxification [15]. Therefore, ferroptosis, a form of cell death characterised by lipid peroxidation and iron-dependent processes, may significantly impact liver transplantation injury. This study focused on investigating the role of ferroptosis in liver IR injury, providing insights into disease mechanisms and identifying potential therapeutic targets to improve patient outcomes.

During cold preservation, the liver is deprived of oxygen and nutrients, leading to decreased ATP production and compromising cell viability and function [16, 17]. However, cold preservation also causes mitochondrial enlargement and membrane damage, resulting in the accumulation of ROS that causes oxidative damage to cellular components [17]. Several studies showed that oxidative stress was elevated in patients undergoing liver transplantation, associated with an increased risk of complications such as graft failure and infection [18, 19]. Reperfusion injury, characterised by the sudden influx of oxygen molecules and nutrients into previously ischaemic tissue, contributes to graft dysfunction and failure by causing additional cellular injury and inflammation [20, 21].

Dex has been shown to protect the liver against IR injury by reducing cellular oxidative stress. This is achieved by reducing the activity of ROS-producing enzymes such as NADPH oxidase and xanthine oxidase, while increasing the activity of antioxidant enzymes such as superoxide dismutase (SOD) and catalase, which act to neutralise ROS [22–24]. However, the molecular mechanisms of noble gases, such as Xe and Ar, in protecting against liver IR injury remain largely undefined. It has been proposed that noble gases may play a role in opening mitochondrial ATP-sensitive K (mitoKATP) channels, thus enhancing mitochondrial function and reducing ROS production [25]. Our study showed that the combination of Dex with noble gases, particularly Xe or Ar, had a stronger protective effect in reducing oxidative stress and regulated cell death, including apoptosis, necroptosis, and ferroptosis, in long-term cold preserved DCD porcine livers.

Our data indicated that hypothermic hypoxia reoxygenation significantly increased TXNIP expression; however, Dex and Ar significantly reduced TXNIP expression and translocation to mitochondria, while increasing thioredoxin expression. TXNIP and thioredoxin are closely related proteins that regulate cellular redox homoeostasis [26]. Thioredoxin is a ubiquitous oxidoreductase critical for maintaining cellular redox balance, catalysing the reduction of disulfide bonds in proteins, and reducing ROS levels in cells [27]. In contrast, TXNIP is shuttled to the mitochondria under oxidative stress; high levels of TXNIP inhibit thioredoxin redox activity, leading to increased hydrogen peroxide levels within the mitochondria. Hydrogen peroxide is a potent oxidising agent that damages proteins, lipids, and DNA within the mitochondria, triggering cell death [28]. Therefore, the inhibition of TXNIP expression and translocation decreases hydrogen peroxide production, oxidative damage, and mitochondrial dysfunction. The combination of Dex and Ar showed potent protection for liver grafts associated with IR injury by reducing TXNIP-mediated oxidative stress and maintaining mitochondrial function.

Our study also revealed that the combination of Dex and Xe or Ar significantly reduced glycogen depletion and hepatocyte reticulin framework collapse. The lack of oxygen during cold preservation activates glycolysis, which is essential for cell survival [29]; regulated cell death may occur if glycolysis is inhibited due to glycogen deficiency [30]. Since most donors are cadaveric, optimising their nutritional status before transplantation through pretreatment is practically impossible. Therefore, reducing glycogen depletion is important to maintain hepatocyte survival during cold preservation [29]. Our data showed that supplementation with Dex and noble gases, in particular Ar, significantly reduced hepatocyte glycogen depletion during cold preservation, potentially providing energy to hepatocytes and reducing graft injury. We also found significant damage to the liver reticulin framework during cold preservation. The reticulin fibres, composed of collagen and glycoprotein, provide structural support to liver cells and help maintain liver architecture [31]. Our work suggested that cold preservation might cause collagen fibres to contract, disrupting the reticulin framework, which led to loss of liver function and increased risk of post-transplantation complications. The combination of Dex with Xe or Ar may help maintain the integrity of the reticulin framework by increasing collagen and glycoprotein content and reducing cell death.

Our study has several limitations. Firstly, DCD livers were only cold preserved without reperfusion during engraftment that mainly causes ROS generation and cell injury. However, the hypothermic hypoxia reoxygenation model to simulate IR injury in an in vitro model was implemented in the current study to investigate the progression of ferroptosis after reperfusion. This is at least in part to compensate this shortfall. Secondly, our study used the cancer cell line HepG2 which are not normal liver cells although it is not nontumorigenic with high proliferation rate and epithelial-like morphology with many differentiated hepatic functions [32]. It has been used in previous studies on liver injury using different models [33, 34]. Even so, in future studies, we will actively consider using a normal liver cell line for further analysis. Lastly, unlike the animal study that evaluated several types of regulated cell death, we only assessed cell viability and ferroptosis in the in vitro model; more cell injurious types should be evaluated in future to demonstrate the impact of Dex and Ar under hypothermic hypoxia reoxygenation condition.

Nevertheless, our study holds significant clinical implications. It is well-recognised that organs procured from donation after brain death (DBD) donors are of higher quality due to the continued circulation at the time of brain death declaration. However, with the growing reliance on marginal or DCD donors to meet the increasing demand for transplantation, it is crucial to enhance graft preservation strategies. DCD organs are particularly vulnerable to the impacts of IR, and effective strategies to mitigate IR injury of DCD organs are urgent for enlarging the donor pool. While this preservation strategy may increase the availability of liver grafts, it is important to note that this study is a proof of concept one. Further validation is required in more clinically relevant settings, such as in large animal (pig) liver transplant models.

In conclusion, our work provided evidence supporting the clinical potential use of Dex and noble gas saturated cold preservation for transplant graft preservation. The combination of Dex with a high concentration of noble gas, especially 50% Ar, showed the most significant benefits in maintaining liver graft morphology and reducing regulated cell death during cold preservation. Clinical translation of Dex and Ar in combination may expand the donor pool by including more liver grafts, shorting waiting times, and improving the quality of life for patients. However, further validation is needed to assess outcomes after engraftment with this preservation strategy.

## Methods

### Ex vivo sample analysis

#### Animals and liver graft cold preservation

In total 5 male 15–17 kg experimental mini pigs were obtained from the Laboratory Animal Centre of Army Medical University, Chongqing, China. The animal study was approved by the Army Medical University Biomedical Ethics Committee (Approval No. AMUWEC20210209) and all procedures followed the ARRIVE guidelines.

The animals fasted for 8 h and were subcutaneously injected with 500 µg/kg midazolam (Pfizer, New York, USA) for sedation [35]. They were then admitted to the animal operating room, receiving non-invasive blood pressure and electrocardiogram monitoring. A peripheral intravenous line was established through the left auricular vein, and 1–2 L/min oxygen was given *via* an animal face mask. After intravenous injection of 20 µg/kg atropine (Mingsheng Medicine, Zhejiang, China), 2 mg/kg propofol (AstraZeneca, Cambridge, UK), and 100 µg fentanyl (Renfu Medicine, Hubei, China) [36], heparin (Dongcheng Pharma, Shandong, China) at 300 IU/kg was administered intravenously 5 min before circulatory death induction [35]. Circulatory death was induced by intravenous injection of 600 mg propofol (AstraZeneca) and then 10 mg atracurium (Hengrui Medicine, Jiangsu, China) [37]. A clamshell thoracotomy was performed to expose the lungs and heart. The left atrial appendage and the precava and postcava were incised for venting blood. 12-F catheters (Byram Healthcare, New York, USA) connecting the infusion apparatus were inserted into the main pulmonary artery and the aorta to flush pulmonary vascular and hepatic vascular [37]. After flushing, the pigs were left at room temperature for in situ warm ischaemia. The liver was extracted 30 min after circulatory death, then placed in the University of Wisconsin solution (UW solution, Bridge to Life Ltd, Columbia, USA) for 24 or 72 h at 4 °C. Non-cold preservation control livers were fixed using 4% paraformaldehyde (Sigma-Aldrich, Poole, UK) immediately after extraction.

#### Cold preservation solutions

Gas mixtures (Heruijia BioMed, Chongqing, China) were prepared in cylinders with 45% O_2_, 5% CO_2_ and balanced with N_2_ in treatment groups as follows:

(1) 45% O_2_, 50% N_2_, and 5% CO_2_; (2) 0.1 nM Dex, 45% O_2_, 50% N_2_, and 5% CO_2_; (3) 0.1 nM Dex, 30% Xe, 45% O_2_, 25% N_2_, and 5% CO_2_; (4) 0.1 nM Dex, 30% Ar, 45% O_2_, 25% N_2_, and 5% CO_2_; (5) 0.1 nM Dex, 15% Xe, 15% Ar, 45% O_2_, 20% N_2_, and 5% CO_2_; (6) 0.1 nM Dex, 50% Xe, 45% O_2_, and 5% CO_2_; (7) 0.1 nM Dex, 50% Ar, 45% O_2_, and 5% CO_2_; (8) 0.1 nM Dex, 25% Xe, 25% Ar, 45% O_2_, and 5% CO_2_.

Gas mixtures were bubbled into 250 mL sterile UW solutions supplemented with 0.1 nM Dex [38] (Sigma-Aldrich, Poole, UK) at 0.5 L/min for 20 min in a gas-washing bottle (ZAUBEE, Chengdu, China).

#### H&E staining

The liver sections were deparaffinised. Nuclei were stained with haematoxylin (Sigma-Aldrich), and the extracellular matrix and cytoplasm were stained with eosin (Sigma-Aldrich). The liver injury criteria were evaluated with cell disruption, nuclei loss, and tissue structure damage. Scores 0: normal, 1: <25%, 2: 25–50%, 3: 50–75%, 4: >75% percentage area.

#### PAS staining

Glycogen was measured in liver sections using a PAS staining kit (Abcam, ab150680, Cambridge, UK) according to the instructions of the manufacturer.

#### Reticulum staining

Liver reticulin fibres were measured in liver sections using a reticulum staining kit (Abcam, ab236473, Cambridge, UK) according to the instructions of the manufacturer.

#### Iron staining

Iron deposition was measured in liver sections using an iron staining kit (Abcam, ab150674, Cambridge, UK) according to the instructions of the manufacturer.

#### OxyIHC oxidative stress assay

Oxidative stress of livers was measured using an OxyIHC™ Oxidative Stress Detection kit (EMD Millipore, S7450, Poole, UK) according to the instructions of the manufacturer.

#### TUNEL assay

The sections were detected with in situ TUNEL assay (Abcam, ab66108, Cambridge, UK. TUNEL^+^ nuclei were visualised by green FITC fluorescence.

#### Immunohistochemistry

For ex vivo fluorescence staining, tissue sections were fixed and permeabilised. They were then blocked with 4% donkey serum (Invitrogen) and incubated with mouse monoclonal anti-GPX4 (1:100, Abcam) or rabbit monoclonal anti-phosphorylated MLKL (1:100, Abcam) overnight at 4 °C. Samples were then washed and incubated with fluorochrome-conjugated secondary antibodies (1:300, Abcam). For in vitro fluorescence staining, the cells were fixed and incubated in 1% normal donkey serum, and subsequently incubated overnight with the 1:100 concentration mouse anti-TXNIP primary antibody (Abcam). Then the second fluorochrome-conjugated secondary antibodies (1:300, Abcam) were incubated. Mitochondria were stained with MitoTracker probes (Sigma-Aldrich, M22426, Poole, UK). Nuclei were stained with DAPI and mounted with VECTASHIELD Mounting Medium (Invitrogen).

### In vitro experiment

#### Cell culture and hypothermic hypoxia reoxygenation injury

Human Hepatoblastoma cells (HepG2 cell line; European Cell Culture Collection, Salisbury, UK) were maintained in DMEM medium (Gibco, Billings, USA) supplemented with 10% fetal bovine Serum (Gibco) and 100 U/mL penicillin-streptomycin (Gibco) at 37 °C in a humidified incubator until confluent. For hypothermic hypoxia reoxygenation injury, the cell culture medium was replaced with the UW cold preservation solution, and the cells were placed at 4 °C for 24 h. Then, the UW solution was replaced with warm fresh medium, and the cells were incubated at 37 °C for another 6, 24 or 48 h.

#### In vitro Dex and Ar treatment

Gas mixture component with 50% Ar, 45% O_2_, and 5% CO_2_ was bubbled into the UW solution supplemented with 0.1 nM Dex for 5 min in an aseptic container. Hep G2 cells were incubated with Dex-50% Ar-saturated UW solution and placed in an air-tight chamber with 50% Ar, 45% O_2_ and 5% CO_2_ at 4 °C for 24 h. After that, the Dex-50%Ar-saturated UW solution was replaced by warm fresh DMEM cell culture medium (Invitrogen), and the cells were incubated for up to 48 h at 37 °C in an incubator.

#### Western Blot

Cell cultures were homogenised to remove cell debris. After being heated, denatured, and loaded onto a NuPAGE 4 to 12% Bis-Tris gel for electrophoresis, the protein extracts (40 g/sample) were transferred to a polyvinylidene difluoride membrane and blocked with 5% BSA in TBST for 1 h at room temperature. Membranes were probed with 1:1000 primary antibodies GPX4 (Abcam), ACSL4 (Abcam), Grp78 (Abcam), HMGB1 (Abcam), catalase (Abcam), thioredoxin (Abcam), SOD1 (Abcam), TXNIP (Abcam) in TBST overnight at 4 °C, followed by HRP-conjugated secondary antibody for 1 h (Cell Signalling, Technology). The loading control was the constitutively expressed protein, Glyceraldehyde 3-phosphate dehydrogenase (GAPDH, Cell Signalling, Technology) or Smooth muscle actin (SMA, Cell Signalling, Technology). The blots were visualised using improved chemiluminescence (Santa Cruz, Dallas, USA).

#### Membrane lipid labelling

The cells were incubated with DilC-AM dye (DilC-AM stain kit, D3911, Paisley, UK) at 5 μl/1 mL in medium for 20 min at 37 °C in the dark. Then, the cells were washed and fixed.

#### Crystal violet staining

The cells were fixed with ice-cold 100% methanol (Sigma). A 0.5% crystal violet solution in 25% methanol (Sigma) was added, and the cells were incubated for 10 min.

### Statistical analysis

All numerical data were expressed as mean ± standard deviation (SD) or median with interquartile range if not normally distributed as appropriate and presented with a scatter plot. Data were analysed using the two-tailed Student’s t-test or analysis of variance (ANOVA) followed by the Turkey test for comparisons where appropriate (GraphPad Prism 5.0, GraphPad Software). A *p* value < 0.05 was considered to be of statistical significance.

### Supplementary information

Original Data File

## References

[1] Black CK, Termanini KM, Aguirre O, Hawksworth JS, Sosin M (2018). Solid organ transplantation in the 21(st) century. Ann Transl Med.

[2] Cappadona R, De Giorgi A, Di Simone E, Di Muzio M, Di Muzio F, Di Muzio F (2020). Infodemiology of solid organ transplantation: relationship to the Global Observatory on Donation and Transplantation data. Eur Rev Med Pharmacol Sci.

[3] Minambres E, Suberviola B, Dominguez-Gil B, Rodrigo E, Ruiz-San Millan JC, Rodriguez-San Juan JC (2017). Improving the outcomes of organs obtained from controlled donation after circulatory death donors using abdominal normothermic regional perfusion. Am J Transplant.

[4] Saeb-Parsy K, Martin JL, Summers DM, Watson CJE, Krieg T, Murphy MP (2021). Mitochondria as therapeutic targets in transplantation. Trends Mol Med.

[5] Pizzino G, Irrera N, Cucinotta M, Pallio G, Mannino F, Arcoraci V (2017). Oxidative stress: harms and benefits for human health. Oxid Med Cell Longev.

[6] Birben E, Sahiner UM, Sackesen C, Erzurum S, Kalayci O (2012). Oxidative stress and antioxidant defense. World Allergy Organ J.

[7] Shi S, Verstegen MMA, Mezzanotte L, de Jonge J, Lowik C, van der Laan LJW (2019). Necroptotic cell death in liver transplantation and underlying diseases: mechanisms and clinical perspective. Liver Transpl.

[8] Patel T, Gores GJ (1998). Apoptosis in liver transplantation: a mechanism contributing to immune modulation, preservation injury, neoplasia, and viral disease. Liver Transpl Surg.

[9] Yamada N, Karasawa T, Wakiya T, Sadatomo A, Ito H, Kamata R (2020). Iron overload as a risk factor for hepatic ischemia-reperfusion injury in liver transplantation: Potential role of ferroptosis. Am J Transplant.

[10] Martens A, Ordies S, Vanaudenaerde BM, Verleden SE, Vos R, Verleden GM (2017). A porcine ex vivo lung perfusion model with maximal argon exposure to attenuate ischemia-reperfusion injury. Med Gas Res.

[11] Nespoli F, Redaelli S, Ruggeri L, Fumagalli F, Olivari D, Ristagno G (2019). A complete review of preclinical and clinical uses of the noble gas argon: evidence of safety and protection. Ann Card Anaesth.

[12] Zhou H, Sun J, Zhong W, Pan X, Liu C, Cheng F (2020). Dexmedetomidine preconditioning alleviated murine liver ischemia and reperfusion injury by promoting macrophage M2 activation via PPARgamma/STAT3 signaling. Int Immunopharmacol.

[13] Tufek A, Tokgoz O, Aliosmanoglu I, Alabalik U, Evliyaoglu O, Ciftci T (2013). The protective effects of dexmedetomidine on the liver and remote organs against hepatic ischemia reperfusion injury in rats. Int J Surg.

[14] Rishi G, Subramaniam VN (2017). The liver in regulation of iron homeostasis. Am J Physiol Gastrointest Liver Physiol.

[15] Jomova K, Valko M (2011). Importance of iron chelation in free radical-induced oxidative stress and human disease. Curr Pharm Des.

[16] Guibert EE, Petrenko AY, Balaban CL, Somov AY, Rodriguez JV, Fuller BJ (2011). Organ preservation: current concepts and new strategies for the next decade. Transfus Med Hemother.

[17] Hassanein T, Frederick T (2004). Mitochondrial dysfunction in liver disease and organ transplantation. Mitochondrion.

[18] Senoner T, Schindler S, Stattner S, Ofner D, Troppmair J, Primavesi F (2019). Associations of oxidative stress and postoperative outcome in liver surgery with an outlook to future potential therapeutic options. Oxid Med Cell Longev.

[19] Burke A, FitzGerald GA, Lucey MR (2002). A prospective analysis of oxidative stress and liver transplantation. Transplantation.

[20] Zhou J, Chen J, Wei Q, Saeb-Parsy K, Xu X (2020). The role of ischemia/reperfusion injury in early hepatic allograft dysfunction. Liver Transpl.

[21] Monbaliu D, Pirenne J, Talbot D (2012). Liver transplantation using donation after cardiac death donors. J Hepatol.

[22] Zeng X, Wang H, Xing X, Wang Q, Li W (2016). Dexmedetomidine protects against transient global cerebral ischemia/reperfusion induced oxidative stress and inflammation in diabetic rats. PLoS ONE.

[23] Chen X, Chen D, Chen P, Chen A, Deng J, Wei J (2022). Dexmedetomidine attenuates apoptosis and neurological deficits by modulating neuronal NADPH oxidase 2-derived oxidative stress in neonates following hypoxic brain injury. Antioxidants (Basel).

[24] Huang J, Jiang Q (2019). Dexmedetomidine protects against neurological dysfunction in a mouse intracerebral hemorrhage model by inhibiting mitochondrial dysfunction-derived oxidative stress. J Stroke Cerebrovasc Dis.

[25] Spaggiari S, Kepp O, Rello-Varona S, Chaba K, Adjemian S, Pype J (2013). Antiapoptotic activity of argon and xenon. Cell Cycle.

[26] Matsuzawa A (2017). Thioredoxin and redox signaling: roles of the thioredoxin system in control of cell fate. Arch Biochem Biophys.

[27] Arner ES, Holmgren A (2000). Physiological functions of thioredoxin and thioredoxin reductase. Eur J Biochem.

[28] Yoshihara E, Masaki S, Matsuo Y, Chen Z, Tian H, Yodoi J (2014). Thioredoxin/Txnip: redoxisome, as a redox switch for the pathogenesis of diseases. Front Immunol.

[29] Quintana AB, Guibert EE, Rodriguez JV (2005). Effect of cold preservation/reperfusion on glycogen content of liver. Concise review. Ann Hepatol.

[30] Imai H, Matsuoka M, Kumagai T, Sakamoto T, Koumura T (2017). Lipid peroxidation-dependent cell death regulated by GPx4 and ferroptosis. Curr Top Microbiol Immunol.

[31] Orah N, Rotimi O, Abdulkareem FB (2016). The use of special stains in liver biopsy interpretation: Implications for the management of liver disease in Nigeria. Niger J Clin Pract.

[32] Donato MT, Tolosa L, Gomez-Lechon MJ (2015). Culture and functional characterization of human hepatoma HepG2 cells. Methods Mol Biol.

[33] Alwadei N, Rashid M, Chandrashekar DV, Rahighi S, Totonchy J, Sharma A (2023). Generation and characterization of CYP2E1-overexpressing HepG2 cells to study the role of CYP2E1 in hepatic hypoxia-reoxygenation injury. Int J Mol Sci.

[34] Kakizaki M, Yamamoto Y, Nakayama S, Kameda K, Nagashima E, Ito M (2021). Human hepatocyte-derived extracellular vesicles attenuate the carbon tetrachloride-induced acute liver injury in mice. Cell Death Dis.

[35] Kaiser GM, Heuer MM, Fruhauf NR, Kuhne CA, Broelsch CE (2006). General handling and anesthesia for experimental surgery in pigs. J Surg Res.

[36] Bazin JE, Constantin JM, Gindre G (2004). Laboratory animal anaesthesia: influence of anaesthetic protocols on experimental models. Ann Fr Anesth Reanim.

[37] Safaee Fakhr B, Dondossola D, Cavenago M, Zanetti M, Gatti S, Gattinoni L (2016). Deterioration of lung function in a pig model of uncontrolled cardiac death. Transplant Proc.

[38] Chen Q, Liu X, Liu Z, Zhang S, Chen L, Eguchi S (2023). Tackling regulated cell death yields enhanced protection in lung grafts. Theranostics.

