# Supplementary material for: Dexmedetomidine and argon in combination against ferroptosis through tackling TXNIP-mediated oxidative stress in DCD porcine livers
Source: Cell Death Discov. 2024 Jul 11;10:319. doi: 10.1038/s41420-024-02071-7 (PMC11239900; doi:10.1038/s41420-024-02071-7)
Supplement: Supplementary file 1 — Original Data File [file 41420_2024_2071_MOESM1_ESM.pdf]

Full unedited gel for Figure 7A -- GPX4 (MW: 22 kDa)

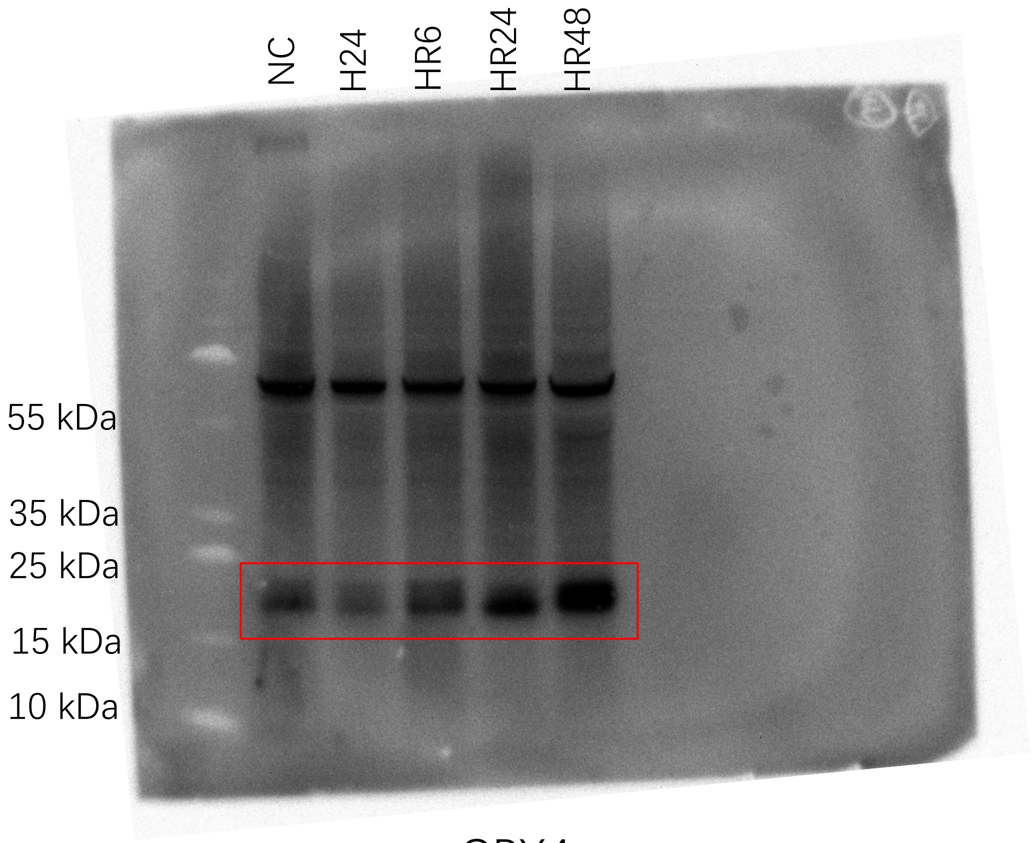

GPX4

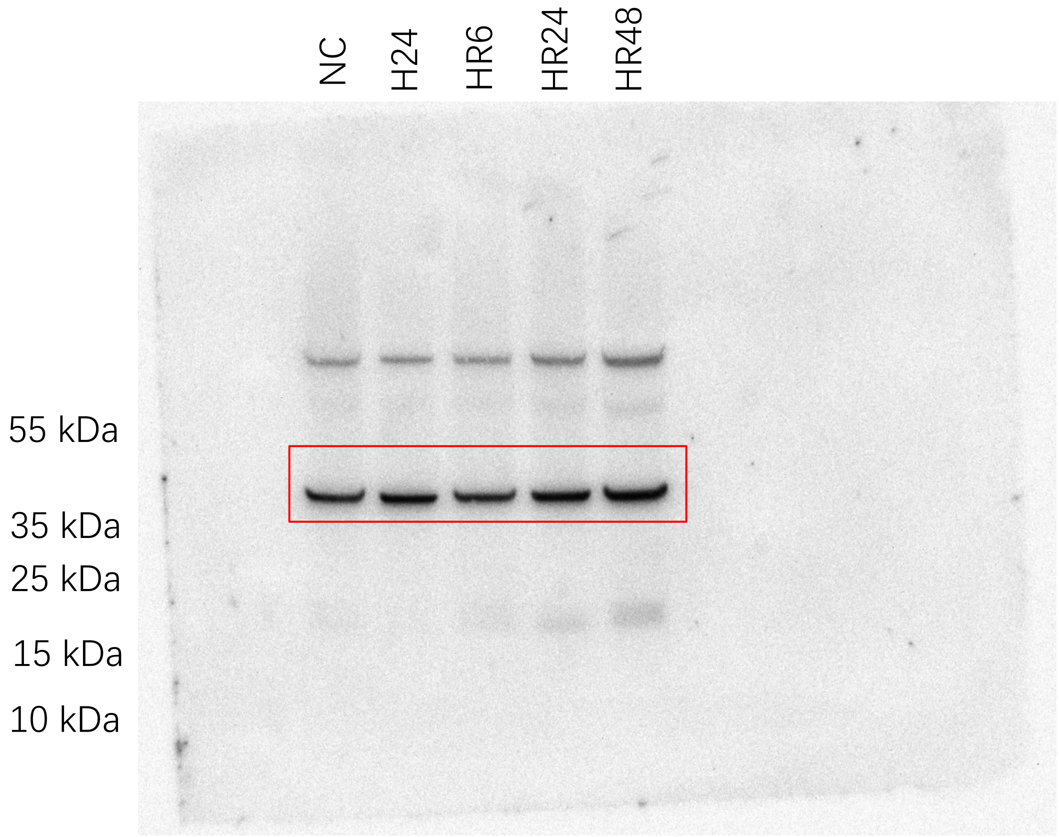

GAPDH

Full unedited gel for Figure 7A -- ACSL4 (MW: 79 kDa)

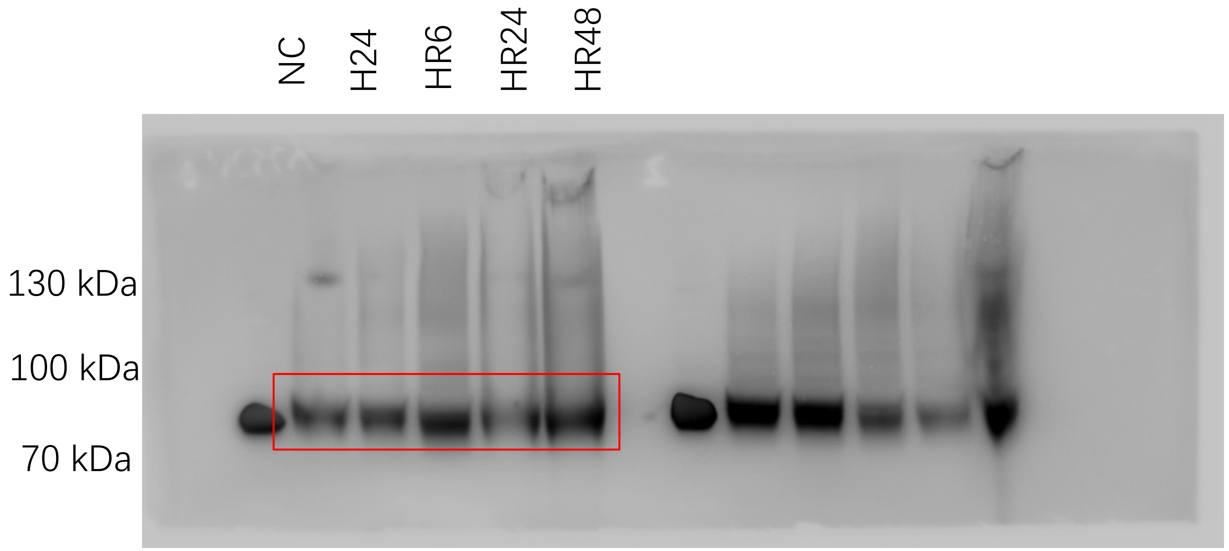

ACSL4

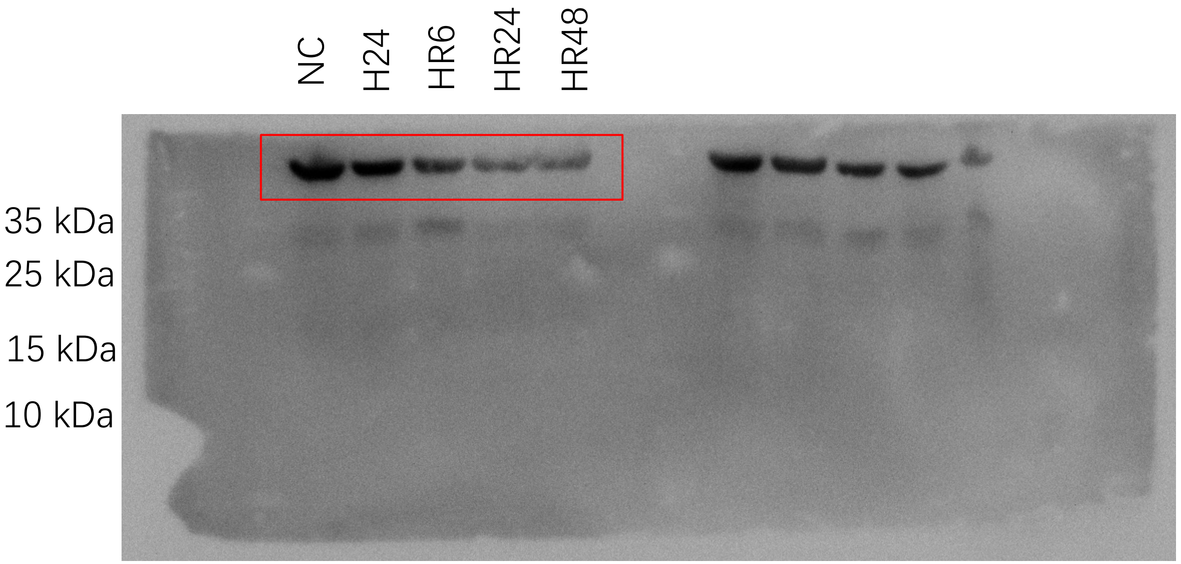

GAPDH

Full unedited gel for Figure 7A -- Grp78 (MW: 78 kDa)

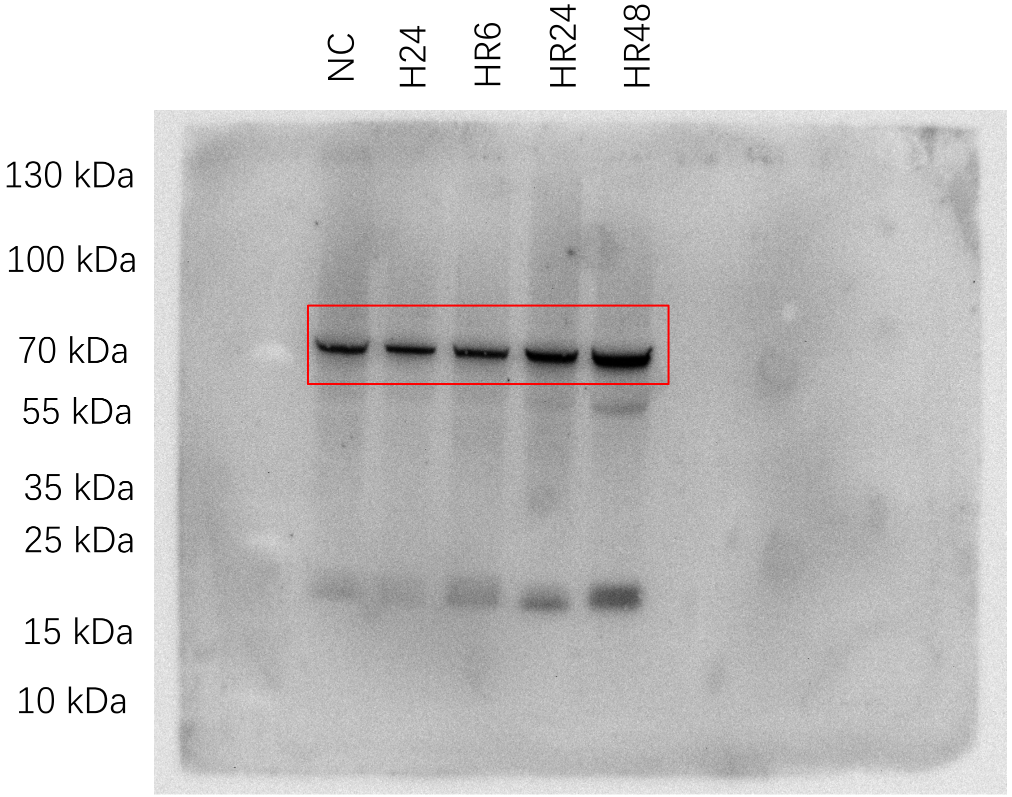

Grp78

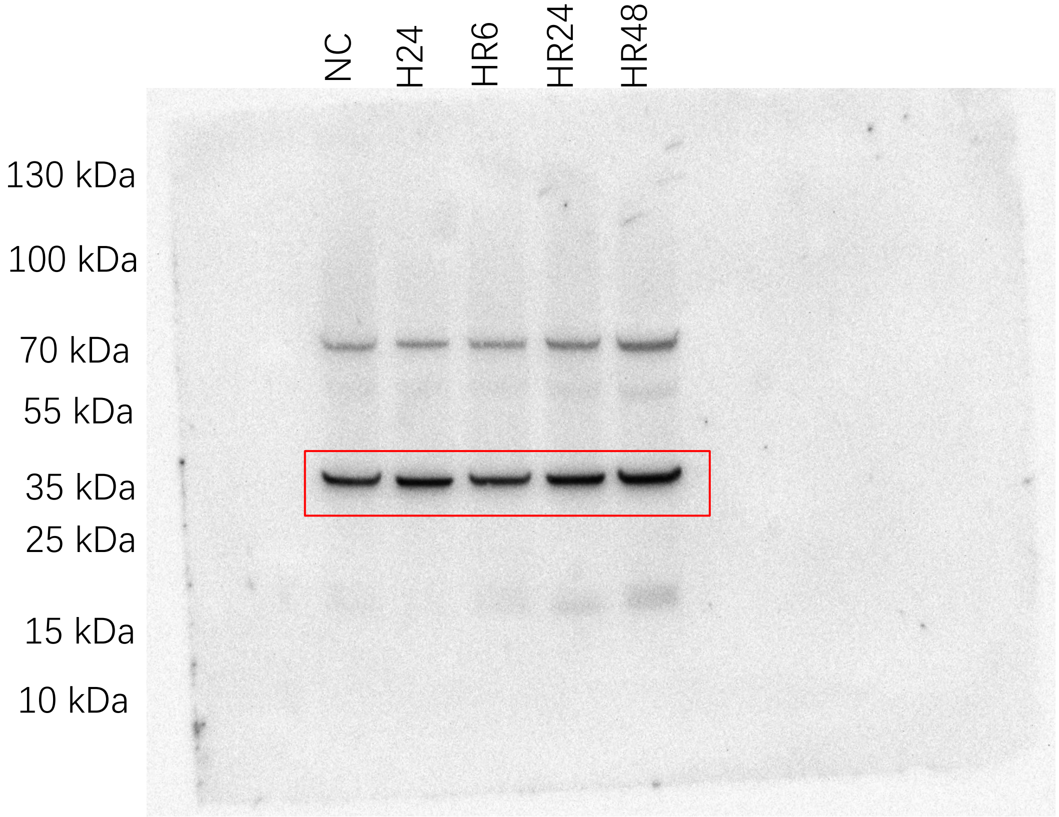

GAPDH

Full unedited gel for Figure 7A -- HMGB1 (MW: 29 kDa)

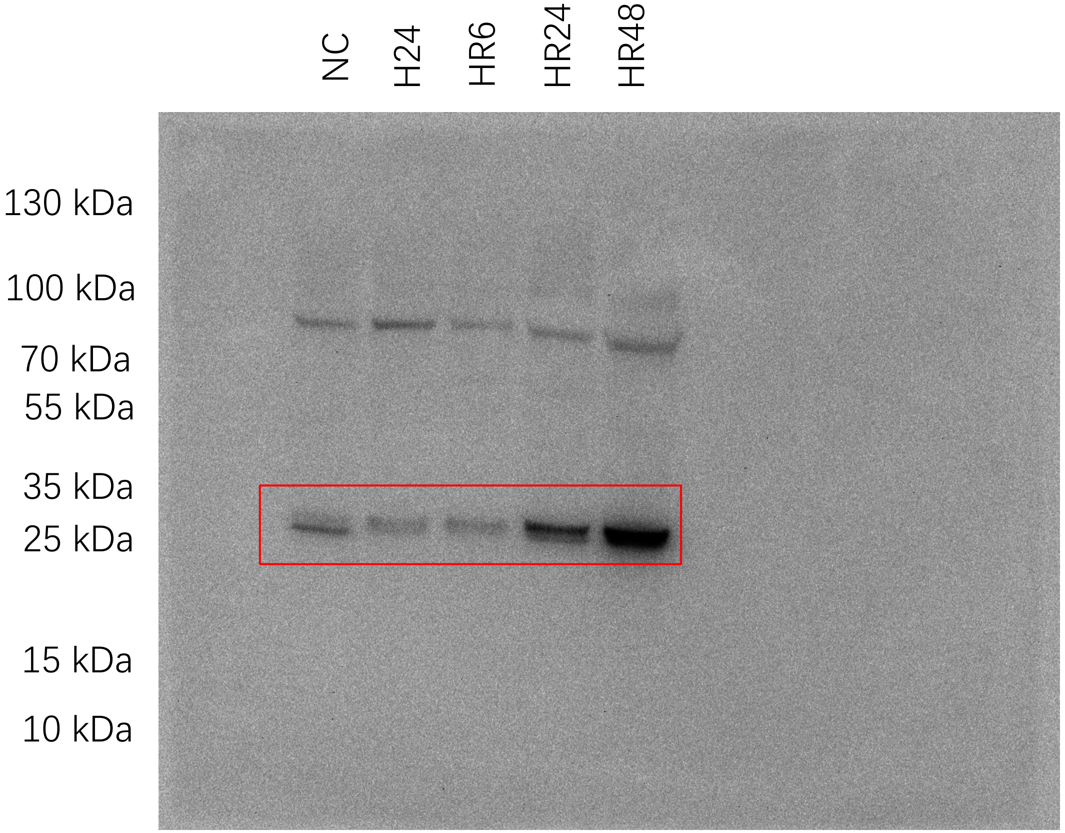

HMGB1

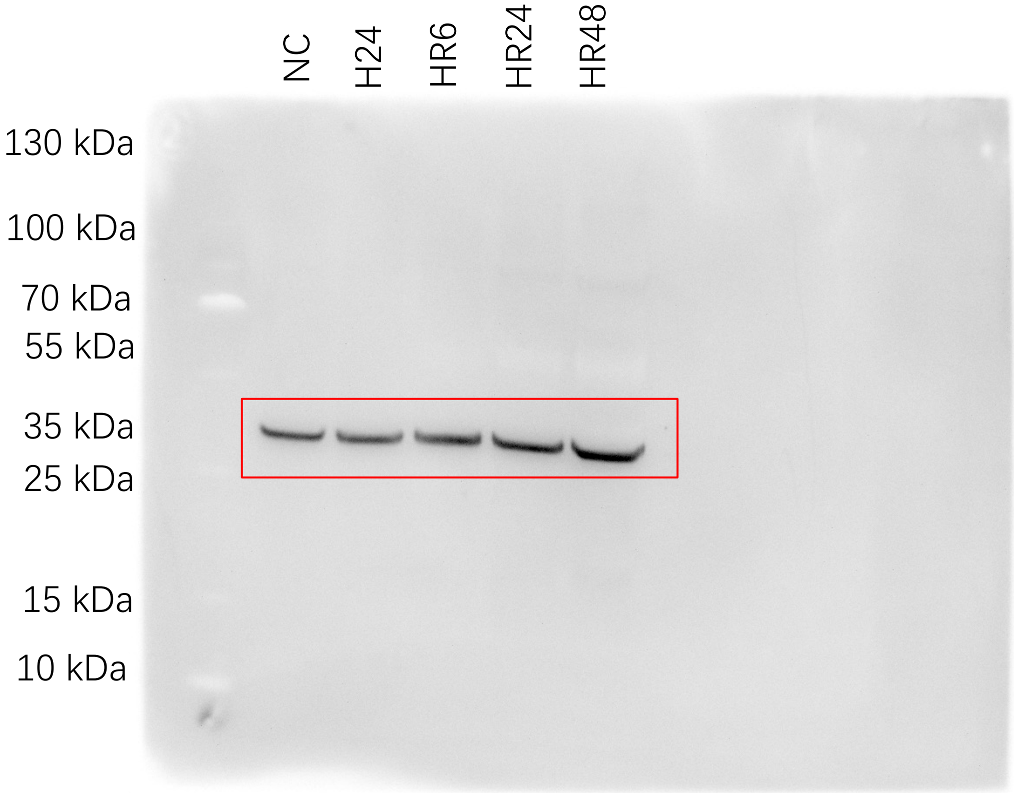

GAPDH

Full unedited gel for Figure 7F -- GPX4 (MW: 22 kDa)

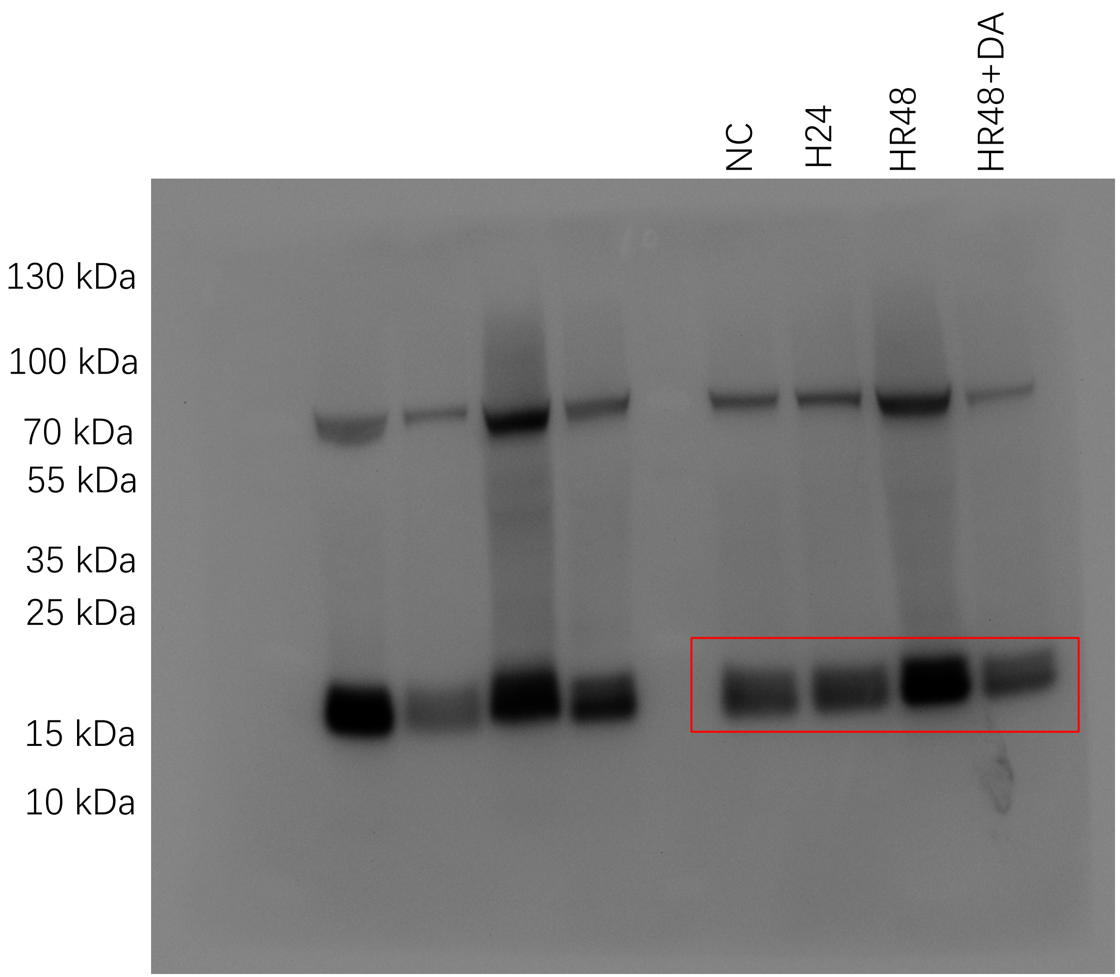

GPX4

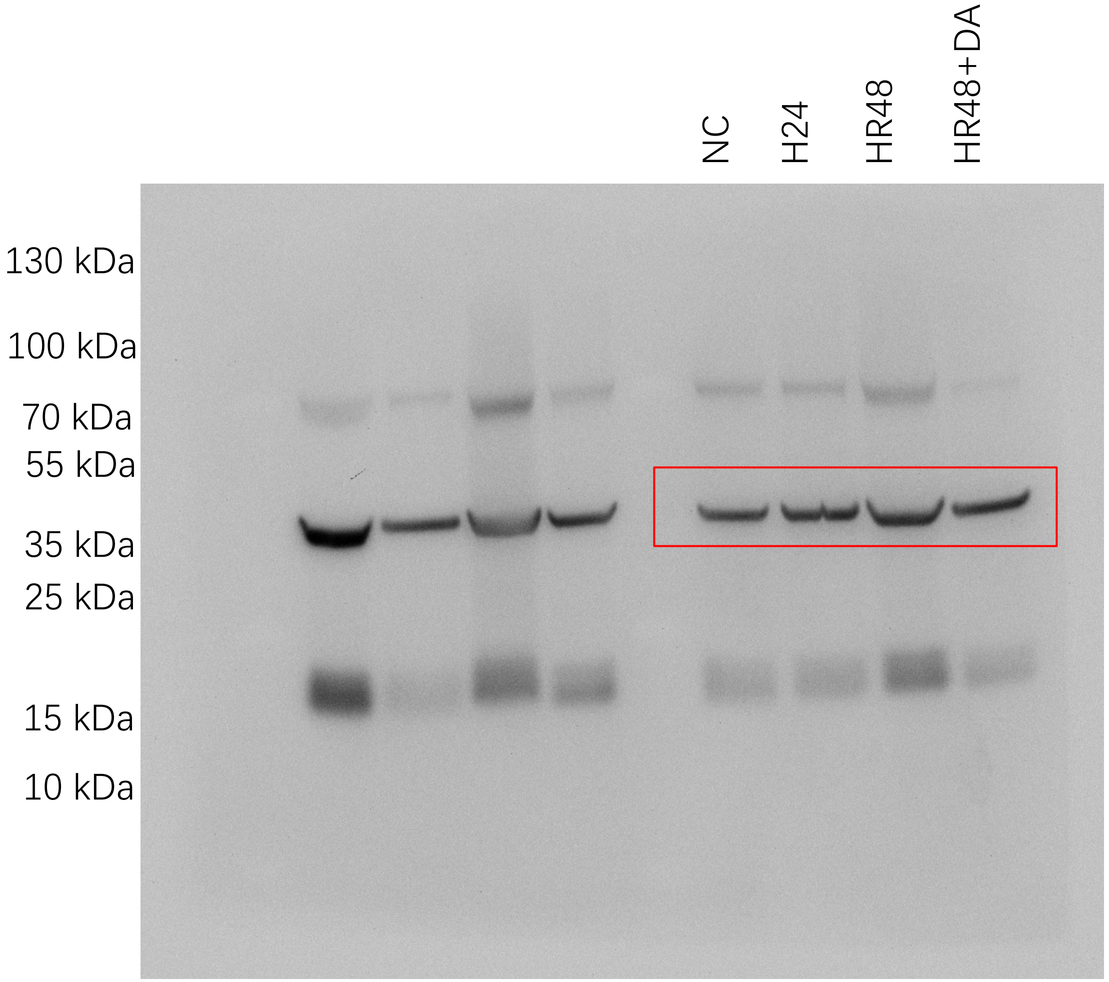

GAPDH

Full unedited gel for Figure 7F -- ACSL4 (MW: 79 kDa)

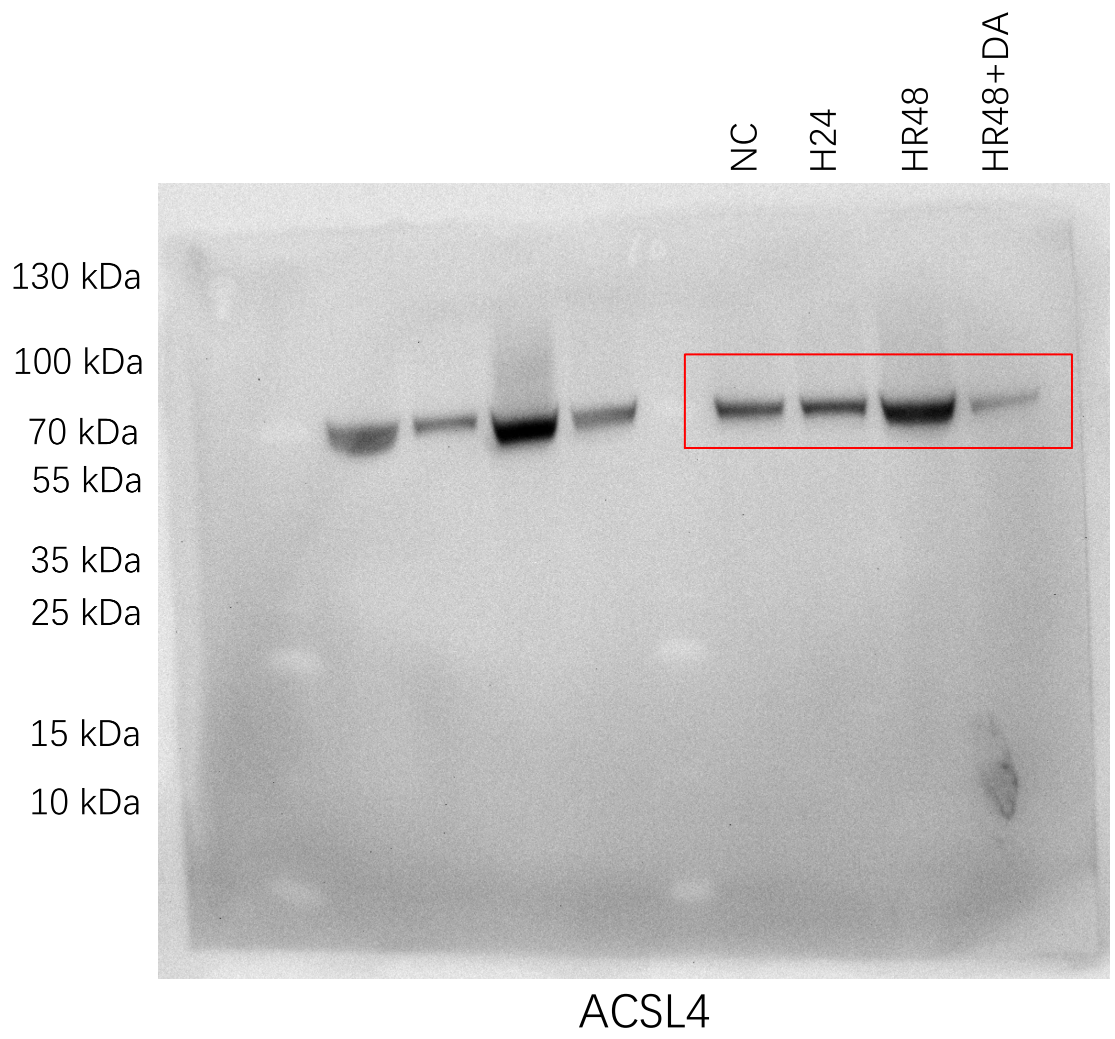

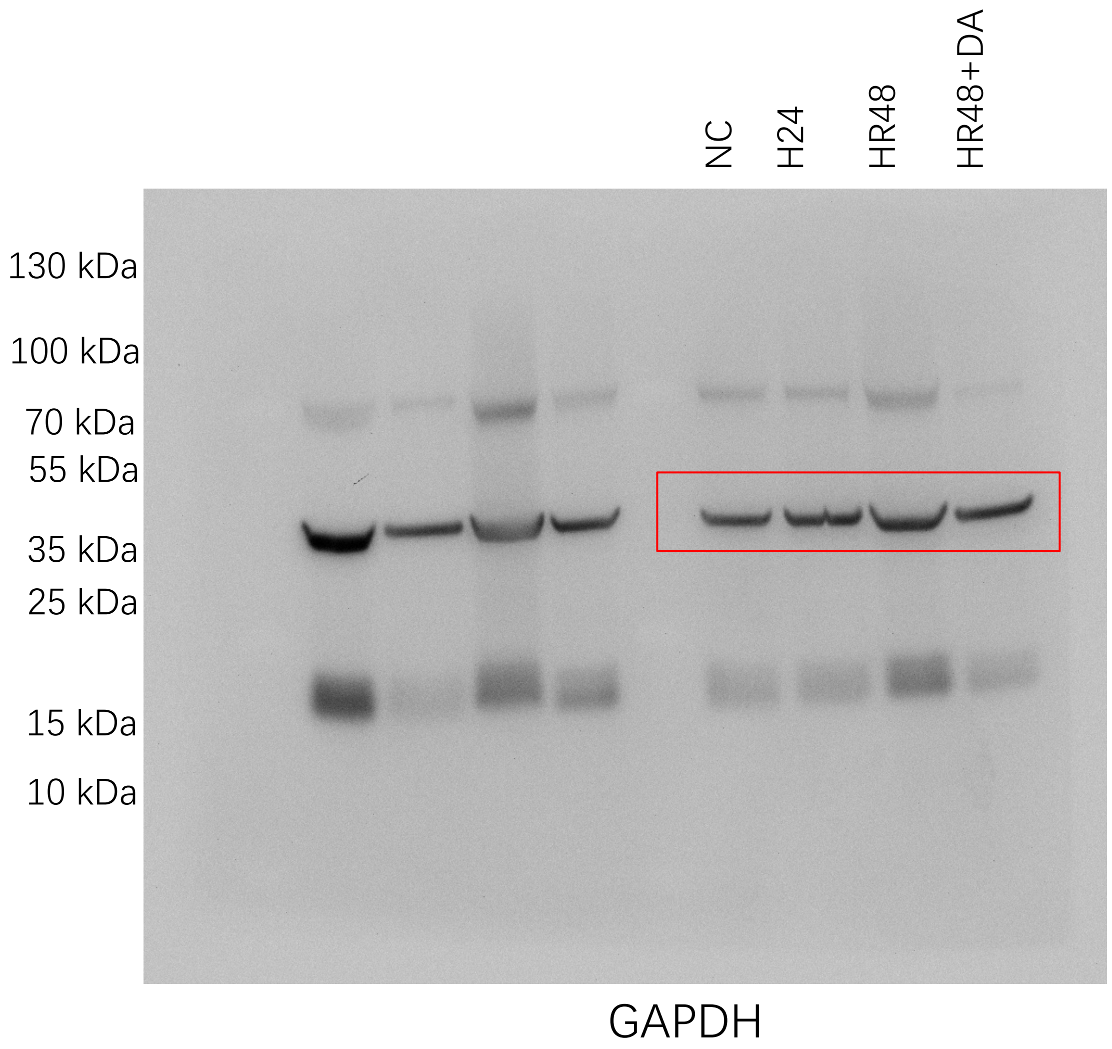

Full unedited gel for Figure 7J -- catalase (MW: 60 kDa)

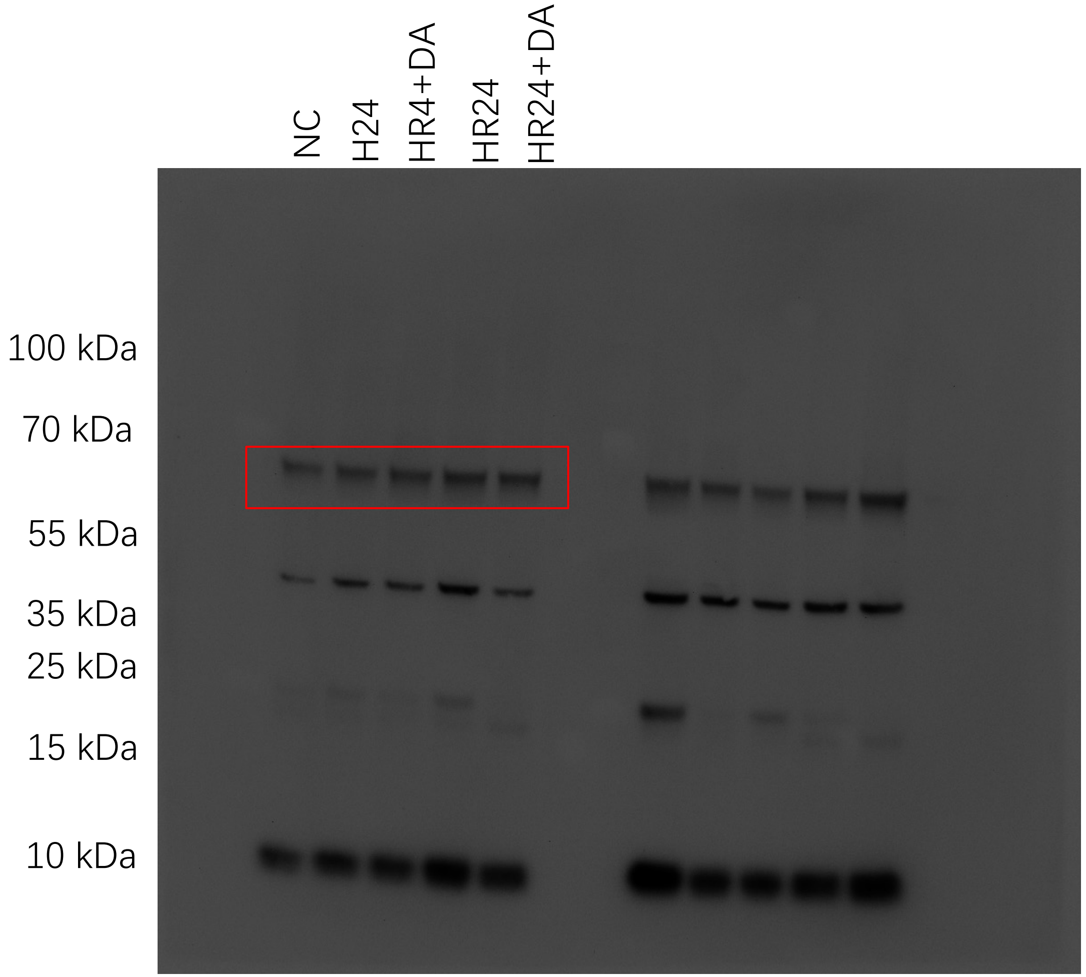

catalase

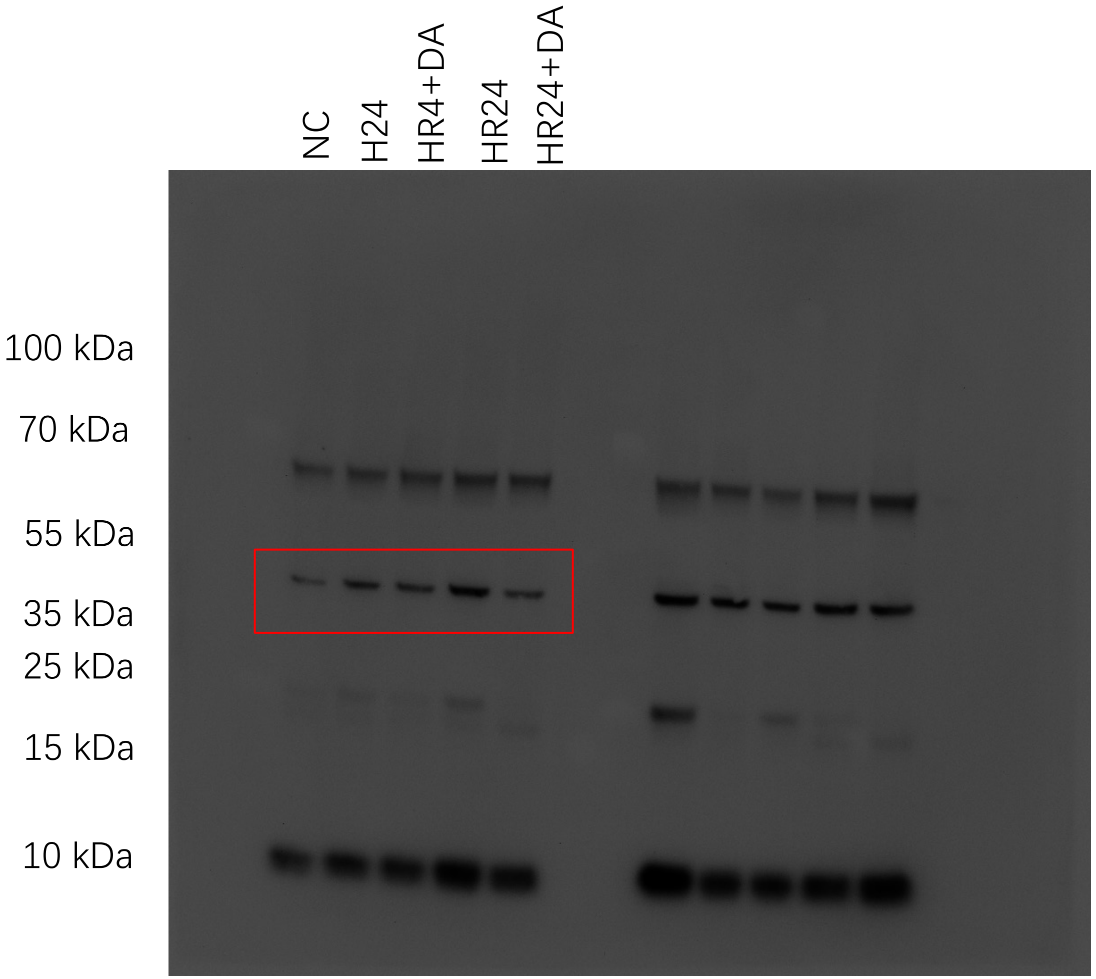

SMA

Full unedited gel for Figure 7J -- thioredoxin (MW: 12 kDa)

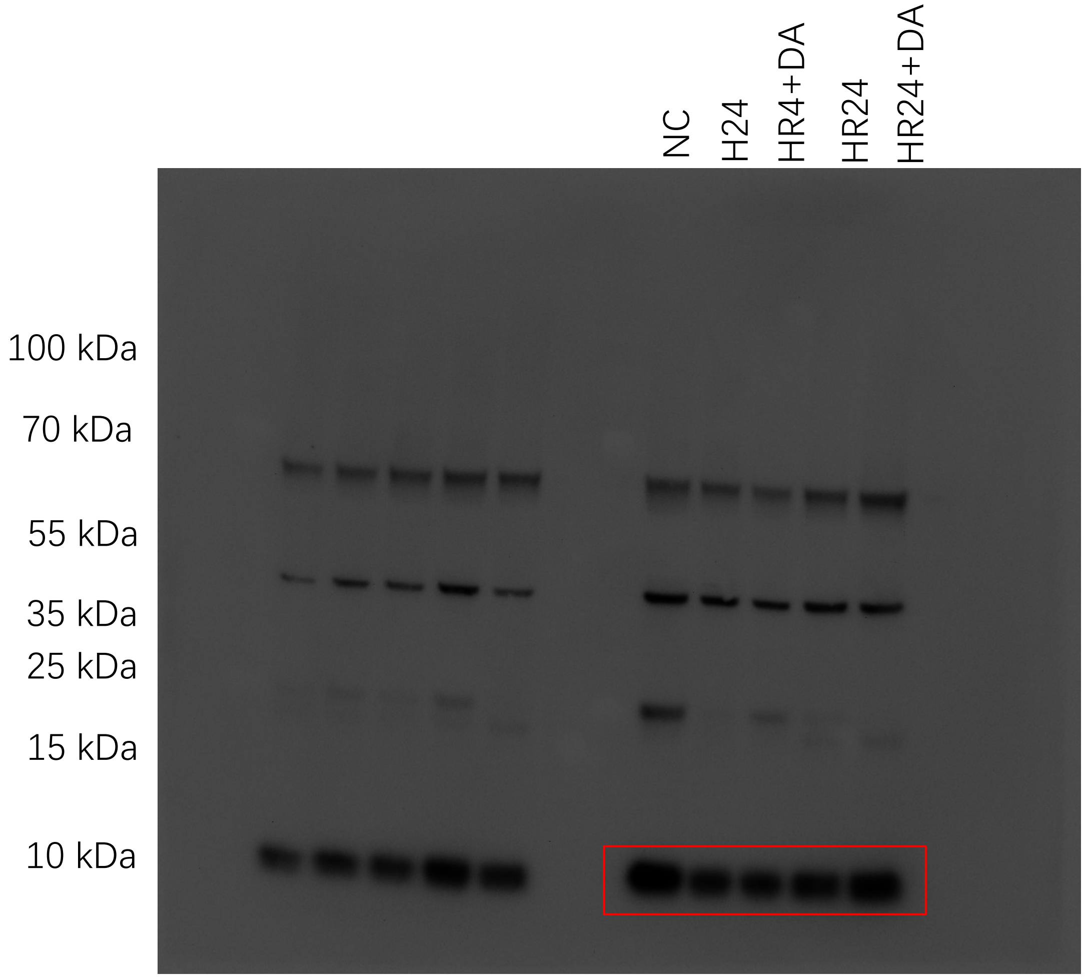

thioredoxin

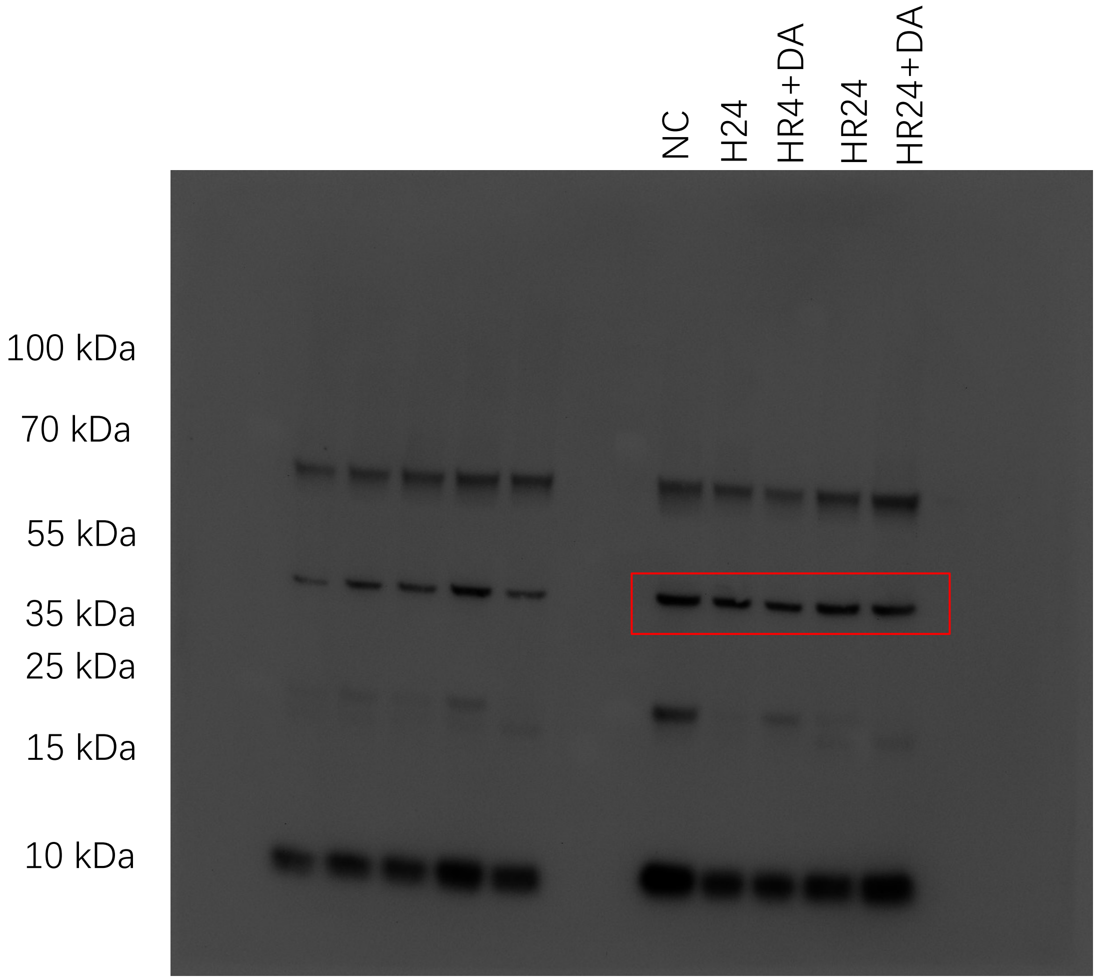

SMA

Full unedited gel for Figure 7J -- SOD1 (MW: 16 kDa)

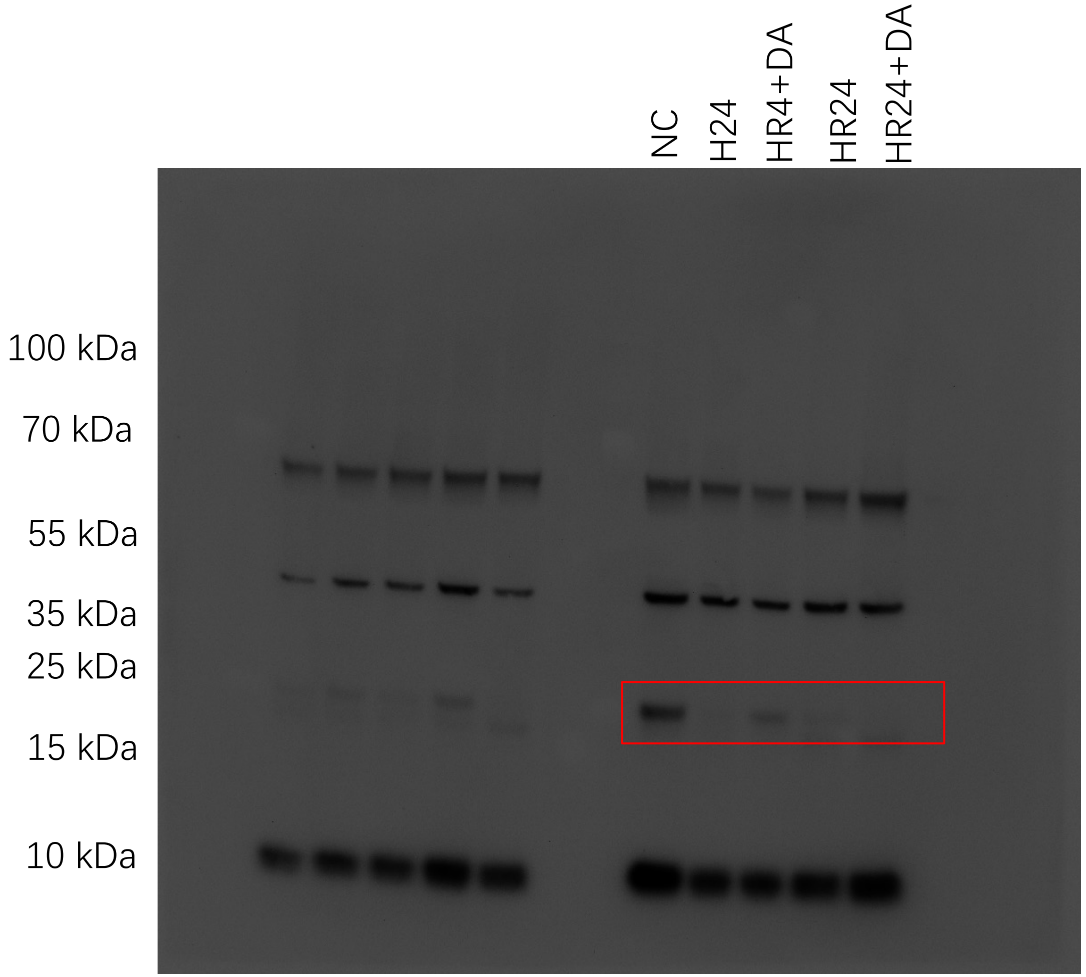

SOD1

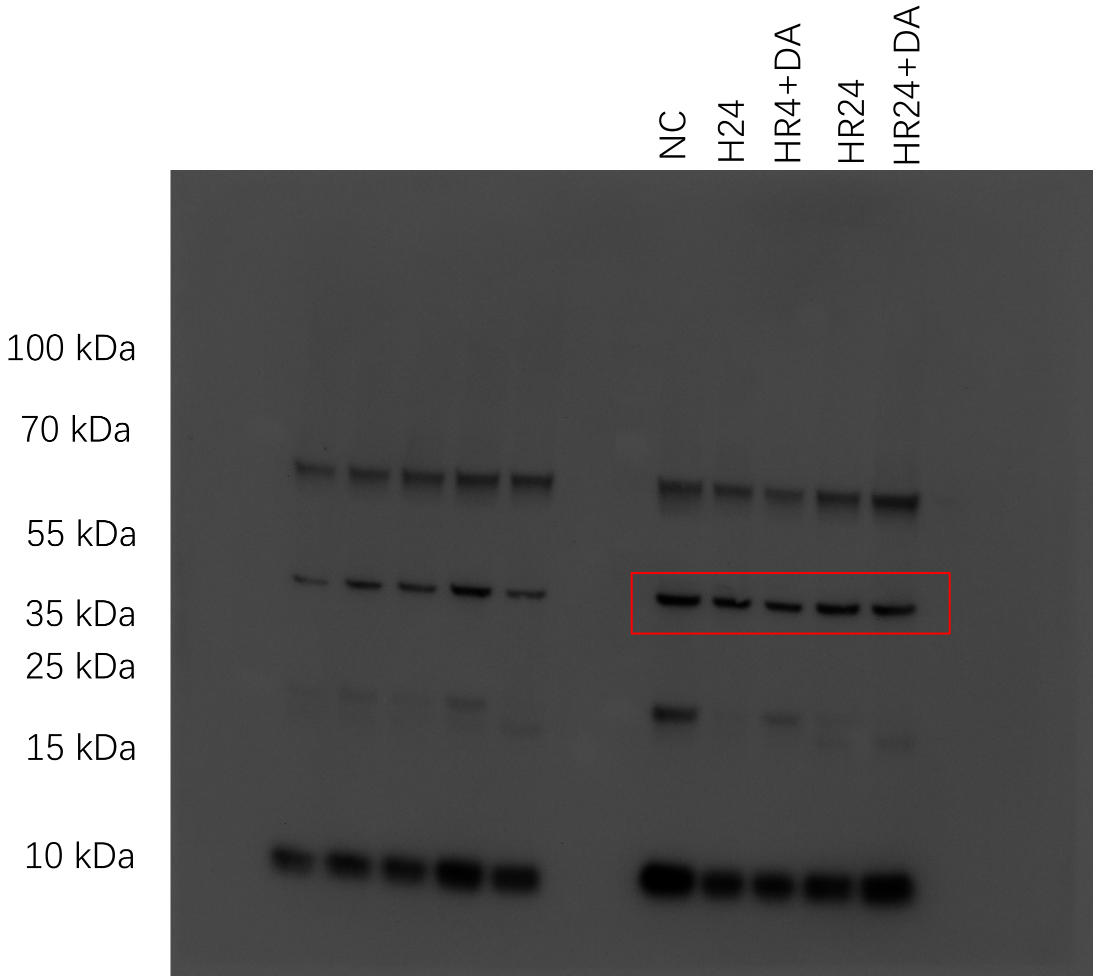

SMA

Full unedited gel for Figure 8B -- TXNIP (MW: 44 kDa)

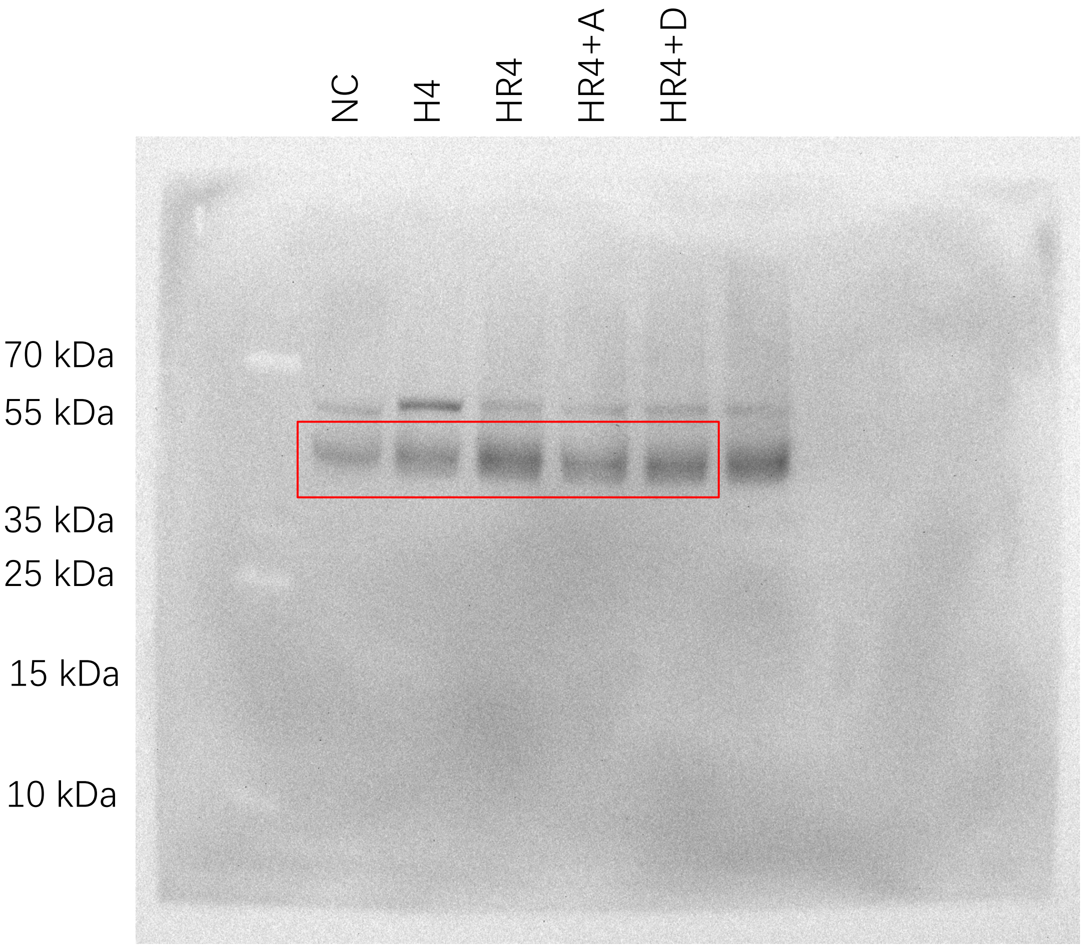

TXNIP

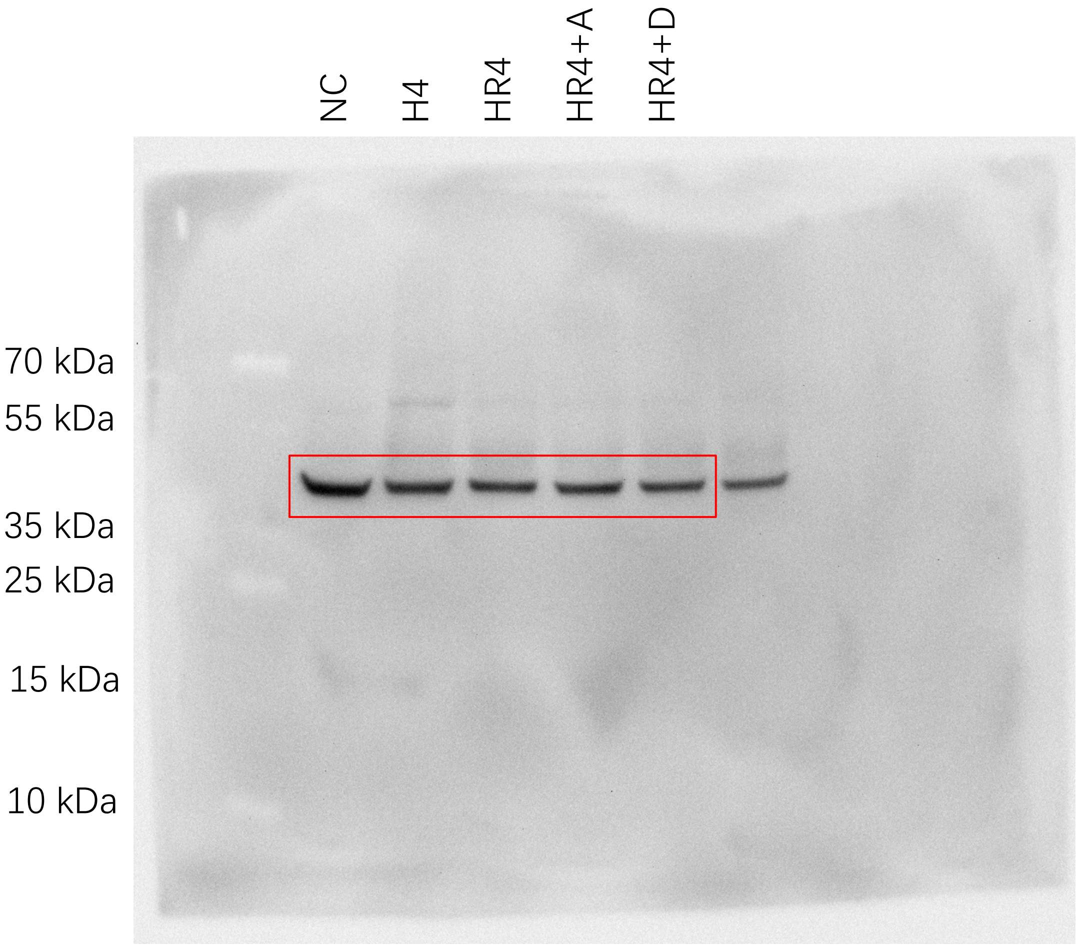

GAPDH
